# Evaluating the effects of coffee consumption on the structure and function of the heart from multiple perspectives

**DOI:** 10.3389/fcvm.2025.1453106

**Published:** 2025-04-15

**Authors:** Xiong-Bin Ma, Yan-Lin Lv, Lin Qian, Jing-Fen Yang, Qian Song, Yong-Ming Liu

**Affiliations:** ^1^ The First Clinical Medical College of Lanzhou University, Lanzhou, Gansu, China; ^2^ The First Clinical Medical College of Guangdong Medical University, Zhanjiang, Guangdong, China; ^3^ Geriatric Cardiovascular Department and Gansu Clinical Research Center for Geriatric Diseases, First Hospital of Lanzhou University, Lanzhou, Gansu, China

**Keywords:** coffee consumption, cardiac structure and function, heart failure, oxidative stress, linkage disequilibrium score regression analysis, Mendelian randomization, causal effect

## Abstract

**Objective:**

To assess the causal relationship between coffee consumption and cardiac structure and function in elderly European populations using multiple genetic methodologies.

**Methods:**

Leveraging genome-wide association study (GWAS) data from elderly European populations, we conducted linkage disequilibrium score regression (LDSC), two-step Mendelian randomization (MR), and colocalization analyses to investigate genetic associations, causal relationships, and mediating effects among these factors. Robustness of findings was verified through comprehensive sensitivity analyses.

**Results:**

LDSC regression analysis revealed positive genetic correlations between coffee consumption and cardiac parameters, excluding left ventricular (LV) ejection fraction and right ventricular (RV) ejection fraction. MR results demonstrated favorable associations between increased coffee consumption and cardiac parameters. After applying the Bonferroni adjustment to IVW analysis, as coffee consumption increased by each 1-cup/day, LV end-diastolic volume increased (*β* = 0.128; 95% CI: 0.043–0.212; *P* = 0.002), an increase in LV end-systolic volume (*β* = 0.143; 95% CI: 0.053–0.232; *P* = 0.001), an increase in RV end-diastolic volume (*β* = 0.200; 95% CI: 0.095–0.305; *P* < 0.001), and an increase in RV stroke volume (*β* = 0.209; 95% CI: 0.104–0.313; *P* < 0.001). Mediation analyses indicated that each 1-cup/day increase in coffee consumption significantly correlated with reduced diastolic blood pressure (DBP) and elevated body mass index (BMI). Notably, higher DBP exhibited inverse associations with ventricular systolic/diastolic functional parameters, whereas increased BMI demonstrated positive associations with these parameters, collectively mitigating age-related ventricular volume loss. No U-shaped associations were detected in linear MR frameworks. Colocalization analyses confirmed shared causal genetic variants between coffee intake and cardiac remodeling phenotypes.

**Conclusions:**

Genetically predicted coffee consumption may counteract age-associated ventricular volume loss in elderly Europeans through dual mediation pathways involving DBP reduction and BMI elevation. These structural adaptations suggest potential cardioprotective mechanisms against senile cardiac atrophy. Future studies should prioritize the integration of coffee consumption into cardiovascular risk assessment frameworks and develop personalized recommendations based on individual health profiles.

## 1 Introduction

Heart failure (HF), a terminal disease characterized by significant clinical heterogeneity, impacts the health of an estimated 64 million individuals globally. It is the primary cause of hospitalization for those aged over 65 in Western countries (1). With the prolongation of life expectancy and the escalating prevalence of cardiovascular diseases (CVDs), the patient population with HF is on a continual rise, thereby amplifying the societal burden. Coffee is one of the world's most popular beverages, holding an important position in people's daily lives. As the pace of modern life speeds up, coffee consumption is rising (2). A 12.5-year longitudinal cohort study, encompassing 450,000 participant instances, indicates that the regular intake of 2–3 cups of coffee per day, irrespective of its caffeine content or form (ground or instant), may contribute to a decrease in both the incidence and mortality rates of CVDs (3). According to the researchers’ subgroup analysis, coffee consumption relates to HF risk in a U-shaped pattern. Consuming 1–4 cups of coffee daily reduces this risk; however, intake exceeding 6 cups daily escalates the risk (4). This implies that coffee's detrimental effects could surpass its advantages. Similar findings emerged from the Framingham Heart Study (FHS) and Atherosclerosis Risk in Communities (ARIC) cohorts, where each additional daily cup of coffee was associated with an 8%–14% reduction in HF risk (CHS: HR = 0.86/cup; ARIC: HR = 0.98/cup) (5). Mechanistically, coffee polyphenols (e.g., chlorogenic acid) improve endothelial function and reduce systemic inflammation, while caffeine enhances insulin sensitivity—effects that collectively mitigate metabolic drivers of HF (6, 7).

Historically, research on CVDs has predominantly centered on incidence and mortality rates, with findings primarily derived from observational studies. Given the inherent limitations of research design, sample size, and confounding variables, definitive conclusions regarding causality remain elusive. It is noteworthy that alterations in heart structure and function signify early HF. Recent epidemiological investigations imply a potential association between coffee and cardiac parameters (8). However, genetic evidence is still insufficient to clarify whether this association reflects a causal relationship or shared genetic factors. Previous investigations primarily focused on obvious clinical outcomes (such as HF hospitalization), with limited attention to subclinical changes in cardiac structure and function, which are key targets for early prevention strategies. To address these gaps, this study aims to investigate the causal effects of coffee consumption on multidimensional cardiac phenotypes, including left ventricular (LV) and right ventricular (RV) structure and function, while describing potential mediating pathways. This will help identify the direct or indirect pathways through which coffee exerts its effects, and provide crucial evidence for HF prevention strategies.

## 2 Methods

### 2.1 Study design

**Figure 1:**
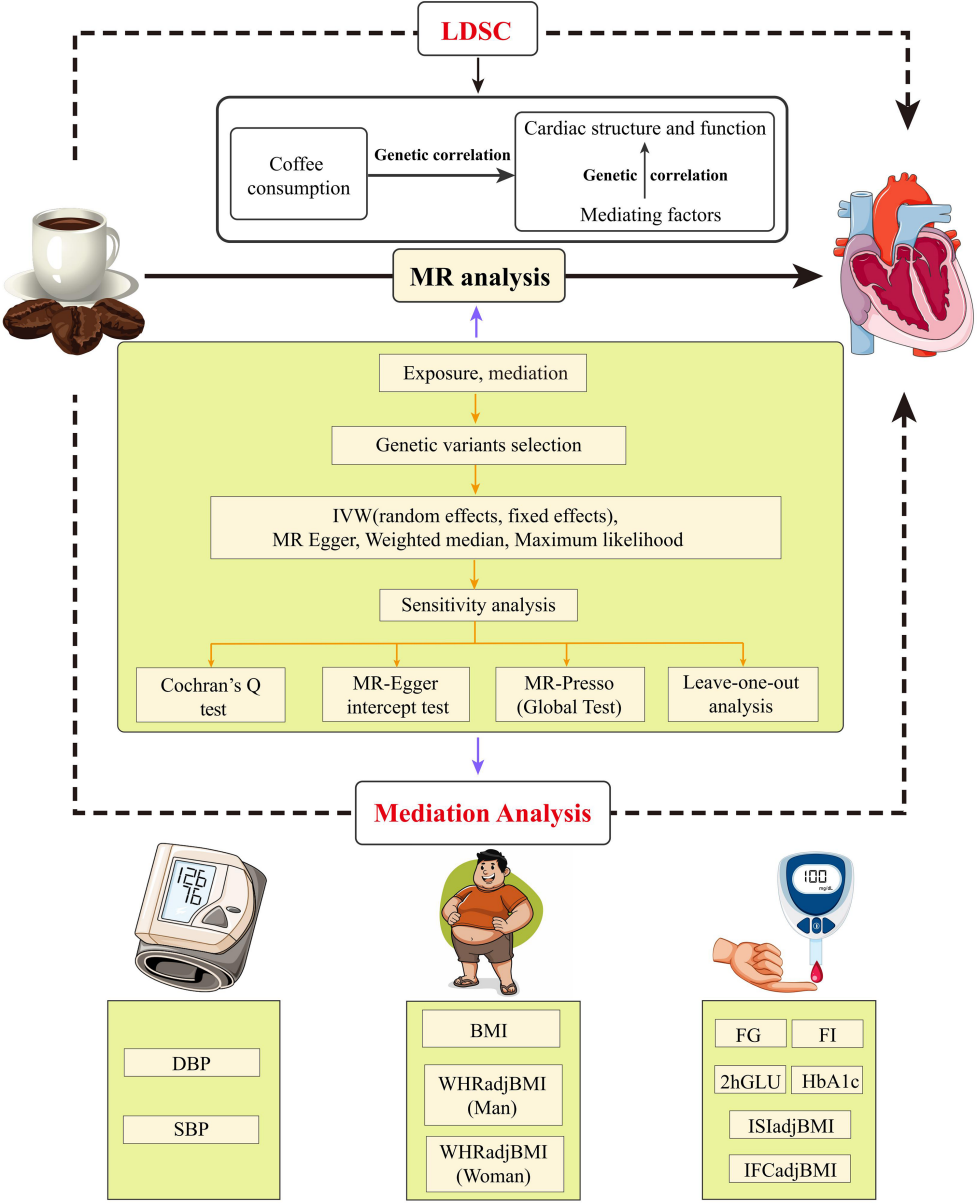
Flow chat and study design. LDSC, linkage disequilibrium score regression; MR, Mendelian randomization; IVW, inverse variance weighting; MR-Presso, MR pleiotropy residual sum and outlier; DBP, diastolic blood pressure; SBP, systolic blood pressure; BMI, body mass index; WHRadjBMI, waist-to-hip ratio adjusted for body mass index; FI, fasting insulin; FG, fasting glucose; 2hGLU, 2h-glucose post-challenge; HbA1c, glycated hemoglobin; ISI, insulin sensitivity index; IFC, insulin fold change.

In this research, we meticulously adhere to the fundamental principles elaborated in the epidemiological observational study report and the STROBE-MR guidelines (9, 10). In Figure 1, the MR study design is explained in detail.

Firstly, we applied Linkage Disequilibrium Score Regression (LDSC) to evaluate the genetic association between coffee intake and cardiac parameters, and then conducted a two-step, two-sample MR analysis to evaluate the causal relationship and mediating factors between the two traits.

### 2.2 Data source

Our study utilized publicly accessible genome-wide association study (GWAS) data, with pertinent ethical approvals documented in the referenced GWAS papers and specific data detailed in Supplementary Table S1. Genetic instruments for coffee consumption were extracted from a GWAS of 448,204 European-ancestry participants in the UK Biobank, where self-reported daily intake (cups/day) was quantified using validated questionnaires. SNPs associated with coffee consumption at a genome-wide suggestive threshold (*P* < 9 × 10^−^⁶) were retained to balance instrument strength and discovery power (11). The investigation considered 11 cardiac metabolic factors as mediators, segregated into anthropometric measurements, glycemic traits, and blood pressure characteristics. We obtained GWAS data on body mass index (BMI) from the European Consortium (*N* = 334,487) (12). The UK Biobank cohort also provided waist-to-hip ratio (WHRadjBMI) adjusted for BMI (males, *N* = 186,825; females, *N* = 219,872) (13), systolic blood pressure (SBP, *N* = 422,713), and diastolic blood pressure (DBP, *N* = 490,469) (14, 15). Data for glycaemic traits encompassing Fasting Glucose (FG, *N* = 281,416), 2-h post-load Glucose (2hGLU, *N* = 281,416), Glycated Hemoglobin (HbA1c, *N* = 281,416), Fasting Insulin (FI, *N* = 281,416), Insulin Sensitivity Index (ISI, *N* = 53,657), and Insulin Fold Change (IFC, *N* = 53,657) (16, 17). The outcome data encompassed GWAS data on LV and RV structural phenotypes derived from cardiac magnetic resonance imaging (MRI) in the UK Biobank cohort (LV end-diastolic volume (LVEDV), LV end-systolic volume (LVESV), LV ejection fraction (LVEF), LV stroke volume (LVSV), RV end-diastolic volume (RVEDV), RV end-systolic volume (RVESV), RV ejection fraction (RVEF), and RV stroke volume (RVSV), *N* = 36,041 and *N* = 29,506, respectively) (18, 19). Excluded from the outcome data samples were patients diagnosed with myocardial infarction or HF, with all samples originating from an elderly demographic.

### 2.3 Genetic IV selection criteria

Qualified instrumental variables (IV) must simultaneously satisfy the following three conditions: (1) The IV is significantly associated with the exposure (*P* < 5 × 10^−8^) (20); (2) The IV is not associated with any confounding factors of exposure-outcome analyses (R^2^ < 0.001, kb = 10,000); and (3) Only the exposure is affected by the IV, not the outcome (21). Additionally, single nucleotide polymorphisms (SNPs) with moderate palindromic alleles should be excluded to avoid bias, and the MR-PRESSO method is employed to identify and remove outlier SNPs. The F-statistic was computed for each SNP to assess its association with exposure, and a F-statistic exceeding 10 is considered to possess sufficient statistical strength (22). The calculation of the F-statistic is done using the following formula: F = R^2^(N−2)/(1-R^2^) (23, 24). In the preceding paragraphs, Kb is the regional scope of chain imbalance, R^2^ is the exposure variance explained by the chosen SNPs, and N is the number of genetic samples per phenotype.

### 2.4 Mediation analysis and mediation effects

In order to investigate the mediating pathways through which coffee consumption influences cardiac structure and function using 11 phenotypes of cardiac metabolism factors, we conducted a two-step MR analysis. Initially, coffee consumption and cardiac metabolism phenotypes were assessed causally. The second step evaluated the causal association between cardiac metabolism factors and the phenotype of cardiac structure and function. Additionally, the mediating effect of each cardiac metabolism factor was calculated using the coefficient product method (25, 26).

### 2.5 Genetic association and direction validation

Given the potential of genetic association analyses between complex traits and diseases to yield etiological insights and aid in the prioritization of potential causal links (27), this study utilized the LDSC method. This approach can robustly assess SNP heritability and the genetic association among complex traits via GWAS data (28). Therefore, we used it to evaluate the genetic association between coffee consumption and heart structure and function. In addition, we use the Steiger test to evaluate the directionality of observed causal relationships (29).

### 2.6 MR analysis

In order to assess the causal relationship, Two Sample MR (version 0.5.7) and MR-PRESSO (version 1.0) in R software (version 4.3.0) were used. Our genetic association analyses were conducted with LDSC software (version 1.0.1). In the main MR analysis, after Bonferroni adjustment, a *P*-value below 0.00625 (0.05/8) is considered statistically significant, while a *P*-value ranging from 0.00625 to 0.05 denotes nominal association.

### 2.7 Statistical analysis for MR

A fundamental method in MR analyses is Inverse-variance weighting (IVW), which evaluates the effect of each SNP through Wald ratios. This technique assigns weights based on the inverse variance of each SNP to ascertain the aggregate causal effect. Upon detecting heterogeneity, the analysis resorts to a random-effects model, in contrast to the fixed-effects model used otherwise (29, 30). To corroborate the robustness of our findings, we additionally utilized the weighted median, MR-Egger regression, and maximum likelihood estimation as auxiliary methods of estimation. Weighted median estimation yields trustworthy results on the condition that over half the weight is contributed by valid genetic instruments (31). MR-Egger regression distinguishes genetic instrument pleiotropy by probing for a non-null intercept in the association between exposure and outcome (32). Maximum likelihood estimation, which minimizes bias by optimizing the likelihood function to deduce parameters of the probability distribution (33). We also employed Cochran's *Q*-test to evaluate heterogeneity and performed a leave-one-out analysis to ascertain if any singular variant is the principal driver of the causal link between exposure and outcome (34). Finally, our application of the MR-PRESSO technique identified and removed outlier SNPs, furnishing refined estimates and pleiotropy analyses post-exclusion of these SNPs (35).

### 2.8 Colocalization analysis

We performed co-localization analyses using the Coloc R package available at [GitHub link]. (default parameters) in order to assess whether coffee consumption and cardiac structure and function share genetic causal variation in genomic regions, defining genomic regions within 50 kb of the instrumental variable with the prior probability of coloc set to the default value, and followed by the use of approximate Bayes factor computation to generate posterior probabilities (PP) for all possible configurations between the two traits: (1) SNPs in the H0 co-localized region are not related to either trait; (2) SNPs in the H1 co-localized region are related to the first trait but not to the second; (3) SNPs in the H2 co-localized region are related to the second trait but not to the first; (4) SNPs in the H3 co-localization region are associated with both traits but not at the same locus; and (5) SNPs in the H4 co-localization region are related to both individual traits and at the same locus (36). Each hypothesized PP is denoted by PP0, PP1, PP2, PP3, and PP4, respectively. In the original published methodology article, a posteriori probability (PH4) of ≥75% was considered to support the existence of common genetic variation within a given genomic region affecting both traits (37).

## 3 Results

### 3.1 Selection of IVs

In compliance with the established screening criteria for IVs, we identified and selected candidate SNPs that fulfill the required qualifications for conducting genetic predictive analyses on phenotypes associated with coffee consumption and cardiac metabolic factors. Moreover, the F-statistics for all the SNPs under consideration exceeded the threshold of 10, indicating robust instrument strength (Supplementary Tables S2–S10).

### 3.2 Effect of coffee consumption on cardiac parameters

**Figure 2:**
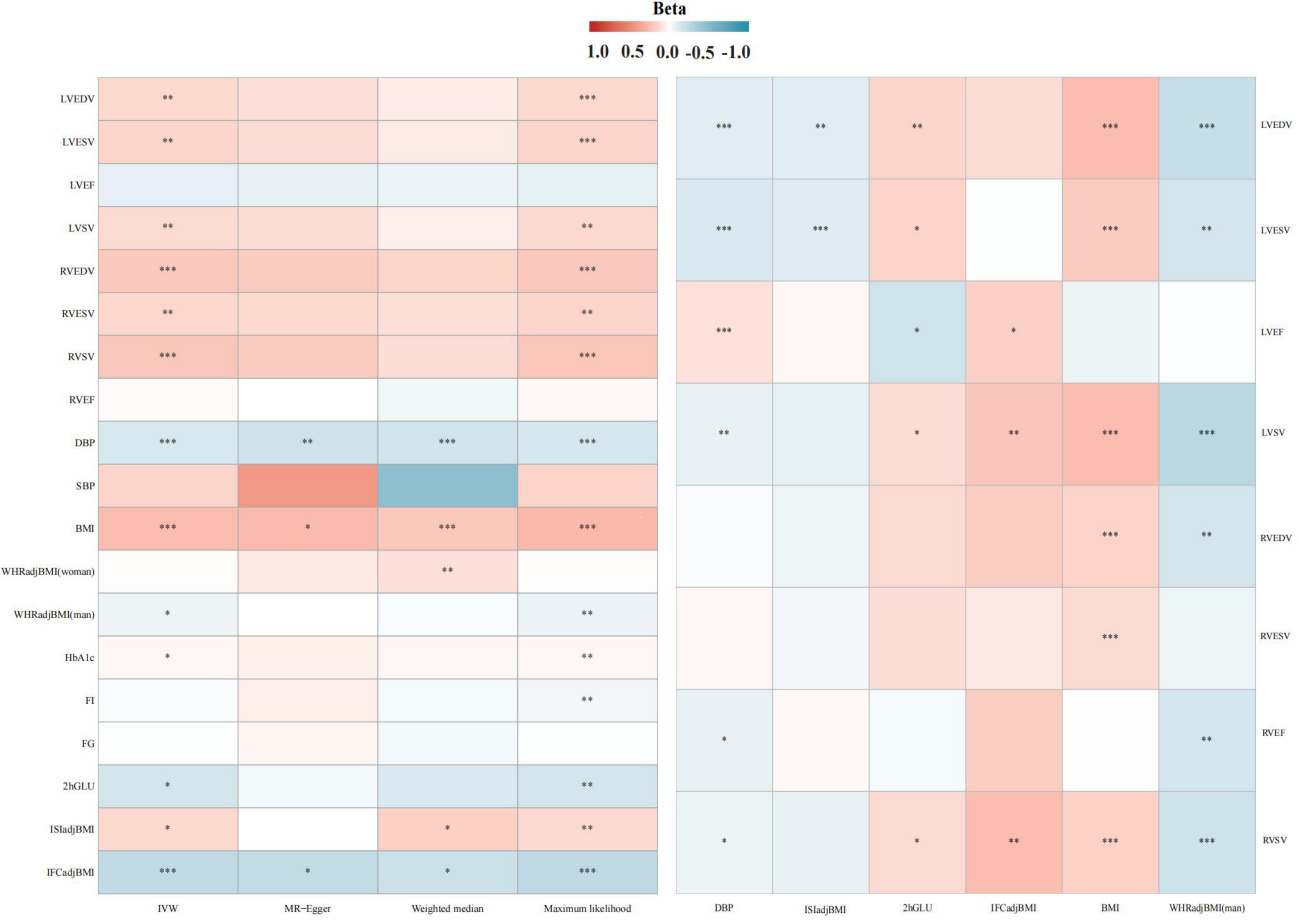
Association heatmap of all Mendelian randomization results. LV, left ventricular; LVEDV, LV end-diastolic volume; LVESV, LV end-systolic volume; LVEF, LV ejection fraction; LVSV, LV stroke volume; RV, right ventricular; RVEDV, RV end-diastolic volume; RVESV, RV end-systolic volume; RVEF, RV ejection fraction; RVSV, RV stroke volume. The explanation of other abbreviations is the same as Figure 1.

**Figure 3:**
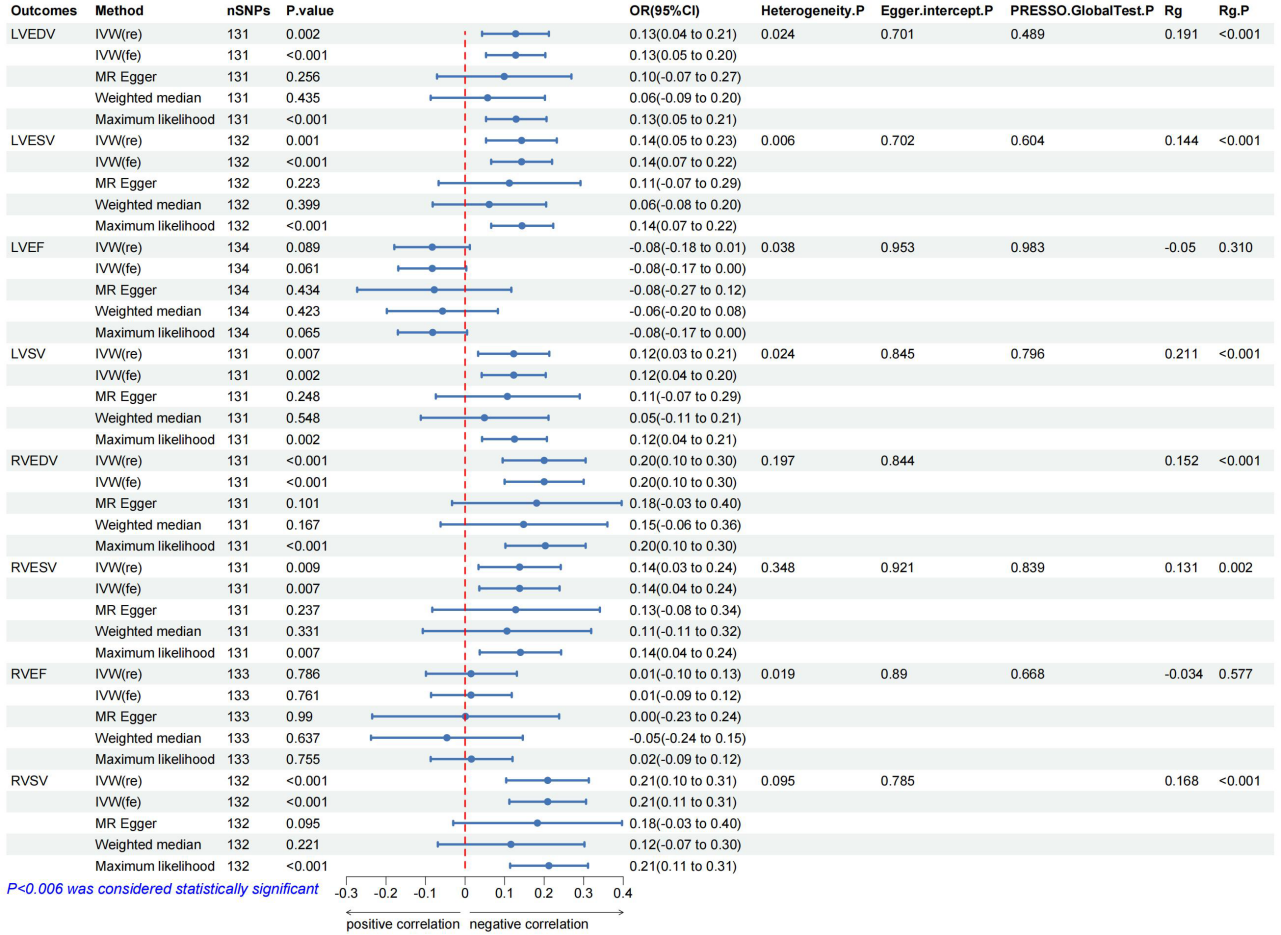
The forest map includes a Mendelian randomization analysis of coffee consumption on cardiac parameters and regression results of the linkage disequilibrium score. Rg, genetic association coefficient.

**Figure 4:**
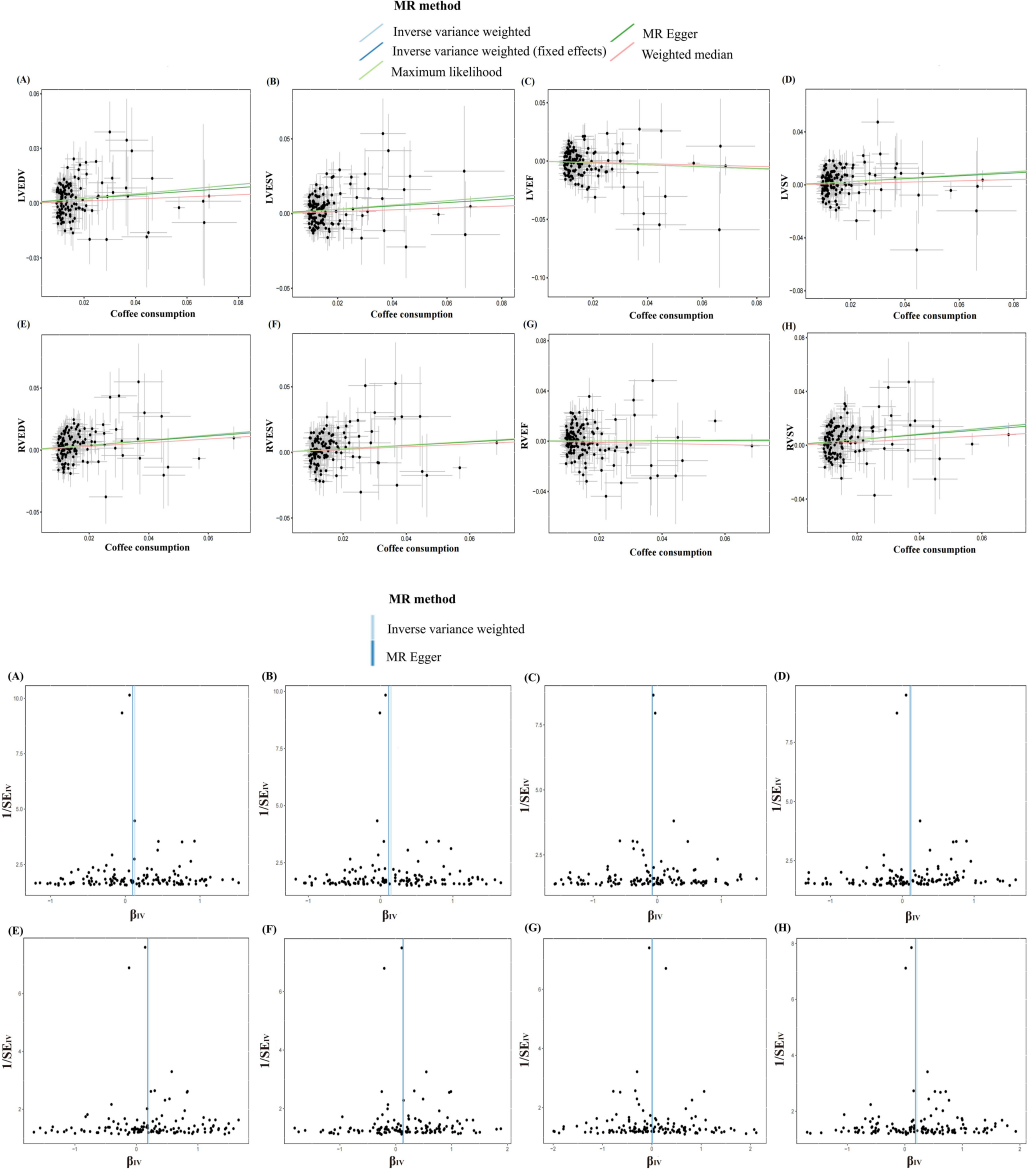
Scatter and funnel plots of the Mendelian randomization analysis of coffee consumption on LVEDV **(A)**, LVESV **(B)**, LVEF **(C)**, LVSV **(D)**, RVEDV **(E)**, RVESV **(F)**, RVEF **(G)**, and RVSV **(H)**, respectively. The explanation of other abbreviations is the same as Figure 2.

In the IVW analysis, after Bonferroni adjustment, each 1-cup/day increase in coffee consumption was significantly associated with an increase in LVEDV (*β* = 0.128; 95% CI: 0.043–0.212; *P* = 0.002), an increase in LVESV (*β* = 0.143; 95% CI: 0.053–0.232; *P* = 0.001), an increase in RVEDV (*β* = 0.200; 95% CI: 0.095–0.305; *P* < 0.001), and an increase in RVSV (*β* = 0.209; 95% CI: 0.104–0.313; *P* < 0.001). Furthermore, each 1-cup/day increase in coffee consumption was nominally associated with an increase in LVSV (*β* = 0.123; 95% CI: 0.033–0.213; *P* = 0.007) and an increase in RVESV (*β* = 0.138; 95% CI: 0.036–0.239; *P* = 0.007) (Figures 2–4, Supplementary Figure S1, Supplementary Table S11).

### 3.3 Effect of coffee consumption on cardiac metabolic factors

In the IVW analysis, each 1-cup/day increase in coffee consumption was significantly associated with a decrease in DBP (*β* = −0.137; 95% CI: −0.184 to −0.089; *P* < 0.001), an increase in BMI (*β* = 0.252; 95% CI: 0.184–0.320; *P* < 0.001), a decrease in WHRadjBMI for males (*β* = −0.065; 95% CI: −0.126 to −0.005; *P* = 0.032), a decrease in 2hGLU (*β* = −0.152; 95% CI: −0.286 to −0.018; *P* = 0.025), and a decrease in IFCadjBMI (*β* = −0.236; 95% CI: −0.343 to −0.128; *P* < 0.001), alongside an increase in ISIadjBMI (*β* = 0.128; 95% CI: 0.015–0.241; *P* = 0.025) (Figure 2, Supplementary Figure S2, Supplementary Table S12).

### 3.4 Effect of cardiac metabolic factors on cardiac parameters

#### 3.4.1 Effect of DBP on cardiac parameters

In the IVW analysis, each 1 SD decrease in DBP was significantly connected with a decrease in LVEDV (*β* = −0.101; 95% CI: −0.154 to −0.047; *P* < 0.001), a decrease in LVESV (*β* = −0.126; 95% CI: −0.181–0.072; *P* < 0.001), a decrease in LVSV (*β* = −0.078; 95% CI: −0.131 to −0.025; *P* = 0.003), a decrease in RVEF (*β* = −0.075; 95% CI: −0.137 to −0.012; *P* = 0.018), and a decrease in RVSV (*β* = −0.065; 95% CI: −0.129 to −0.001; *P* = 0.045), alongside an increase in LVEF (*β* = 0.095; 95% CI: 0.040–0.150; *P* < 0.001) (Figure 2, Supplementary Figure S3, Supplementary Table S13).

#### 3.4.2 Effect of BMI on cardiac parameters

In the IVW analysis, each 1 SD decrease in BMI was significantly connected with an increase in LVEDV (*β* = 0.245; 95% CI: 0.186–0.303; *P* < 0.001), an increase in LVESV (*β* = 0.187; 95% CI: 0.129–0.246; *P* < 0.001), an increase in LVSV (*β* = 0.248; 95% CI: 0.188–0.308; *P* < 0.001), an increase in RVEDV (*β* = 0.153; 95% CI: 0.082–0.224; *P* < 0.001), a decrease in RVESV (*β* = 0.124; 95% CI: 0.053–0.196; *P* < 0.001), and a decrease in RVSV (*β* = 0.160; 95% CI: 0.090–0.230; *P* < 0.001) (Figure 2, Supplementary Figure S4, Supplementary Table S14).

#### 3.4.3 Effect of WHRadjBMI on cardiac parameters in men

In the IVW analysis, each 1 SD decrease in WHRadjBMI (man) was significantly connected with a decrease in LVEDV (*β* = −0.211; 95% CI: −0.308 to −0.115; *P* < 0.001), a decrease in LVSV (*β* = −0.263; 95% CI: −0.364 to −0.162; *P* < 0.001), and a decrease in RVEF (*β* = −0.145; 95% CI: −0.246 to −0.044; *P* < 0.001) (Figure 2, Supplementary Figure S5, Supplementary Table S15).

#### 3.4.4 Effect of 2hGLU on cardiac parameters

In the IVW analysis, each 1 SD decrease in 2hGLU was significantly connected with a decrease in LVEDV (*β* = −0.108; 95% CI: −0.193 to −0.022; *P* = 0.013) and a decrease in LVESV (*β* = −0.109; 95% CI: −0.187 to −0.031; *P* = 0.005) (Figure 2, Supplementary Figure S6, Supplementary Table S16).

#### 3.4.5 Effect of ISIadjBMI on cardiac parameters

In the IVW analysis, each 1 SD decrease in ISIadjBMI was significantly connected with an increase in LVEDV (*β* = 0.143; 95% CI: 0.049–0.238; *P* = 0.002), an increase in LVESV (*β* = 0.149; 95% CI: 0.052–0.246; *P* = 0.002), and a decrease in LVEF (*β* = −0.174; 95% CI: −0.292 to −0.057; *P* = 0.003) (Figure 2, Supplementary Figure S7, Supplementary Table S17).

#### 3.4.6 Effect of IFCadjBMI on cardiac parameters

In the IVW analysis, each 1 SD decrease in IFCadjBMI was significantly connected with an increase in LVEF (*β* = 0.163; 95% CI: 0.008–0.318; *P* = 0.038), and an increase in LVSV (*β* = 0.213; 95% CI: 0.072–0.354; *P* = 0.002) (Figure 2, Supplementary Figure S8, Supplementary Table S18).

### 3.5 Proportion of the mediatory effect of DBP and BMI

We investigated the indirect impact of coffee consumption on cardiac structure and function, mediated through DBP and BMI. Our findings indicate that DBP's mediation effect on LVEDV, LVESV, and RVSV were 0.013 (95% CI: 0.009–0.018; *P* < 0.001), constituting a mediation proportion of 10.8% (95% CI: 7.4%–14.1%); 0.017 (95% CI: 0.012–0.021; *P* < 0.001), accounting for a mediation proportion of 12.1% (95% CI: 8.7%–15.3%); and 0.008 (95% CI: 0.005–0.012; *P* = 0.022), contributing to a mediation proportion of 4.2% (95% CI: 2.3%–6.1%), respectively. In addition, BMI's mediation effect on LVEDV, LVESV, LVSV, RVEDV, RVESV, and RVSV were 0.061 (95% CI: 0.050–0.073; *P* < 0.001), constituting a mediation proportion of 48.2% (95% CI: 39.3%–57%); 0.049 (95% CI: 0.038–0.059; *P* < 0.001), accounting for a mediation proportion of 34.3% (95% CI: 26.8%–41.8%); 0.065 (95% CI: 0.052–0.077; *P* < 0.001), accounting for a mediation proportion of 52.8% (95% CI: 42.8%–62.8%); 0.041 (95% CI: 0.030–0.052; *P* < 0.001), accounting for a mediation proportion of 20.9% (95% CI: 15.4%–26.3%); 0.034 (95% CI: 0.023–0.044; *P* < 0.001), accounting for a mediation proportion of 24.8% (95% CI: 17.3%–32.2%); and 0.043 (95% CI: 0.032–0.054; *P* < 0.001), accounting for a mediation proportion of 20.6% (95% CI: 15.3%–25.8%), respectively (Table 1, Supplementary Table S19).

**Table 1:** The mediation effect of coffee consumption on cardiac parameters via DBP and BMI.

Mediator/Outcome	Total effect	Direct effect A	Direct effect B	Mediation effect	*P*	Mediated proportion (%) (95% CI)
	β (95% CI)	β (95% CI)	β (95% CI)	β (95% CI)		
DBP/LVEDV	0.128 (0.043, 0.212)	−0.137 (−0.184, −0.089)	−0.101 (−0.154, −0.047)	0.013 (0.009, 0.018)	1.00E-03	10.8 (7.4, 14.1)
DBP/LVESV	0.143 (0.053, 0.232)	−0.137 (−0.184, −0.089)	−0.126 (−0.181, −0.072)	0.017 (0.012, 0.021)	2.00E-04	12.1 (8.7, 15.3)
DBP/RVSV	0.209 (0.112, 0.306)	−0.137 (−0.184, −0.089)	−0.065 (−0.129, −0.001)	0.008 (0.005, 0.012)	2.20E-02	4.2 (2.3, 6.1)
BMI/LVEDV	0.128 (0.043, 0.212)	0.252 (0.184, 0.320)	0.245 (0.186, 0.303)	0.061 (0.050, 0.073)	5.00E-08	48.2 (39.3, 57)
BMI/LVESV	0.143 (0.053, 0.232)	0.252 (0.184, 0.320)	0.195 (0.134, 0.257)	0.049 (0.038, 0.059)	4.31E-06	34.3 (26.8, 41.8)
BMI/LVSV	0.123 (0.033, 0.213)	0.252 (0.184, 0.320)	0.258 (0.193, 0.323)	0.065 (0.052, 0.077)	1.14E-07	52.8 (42.8, 62.8)
BMI/RVEDV	0.200 (0.100, 0.300)	0.252 (0.184, 0.320)	0.166 (0.088, 0.244)	0.041 (0.030, 0.052)	4.81E-05	20.9 (15.4, 26.3)
BMI/RVESV	0.138 (0.036, 0.239)	0.252 (0.184, 0.320)	0.136 (0.059, 0.214)	0.034 (0.023, 0.044)	8.00E-04	24.8 (17.3, 32.2)
BMI/RVSV	0.209 (0.112, 0.306)	0.252 (0.184, 0.320)	0.171 (0.096, 0.246)	0.043 (0.032, 0.054)	8.37E-05	20.6 (15.3, 25.8)

'Total effect” indicates the effect of coffee consumption on cardiac parameters, “direct effect A” indicates the effect of coffee consumption on DBP, BMI, “direct effect B” indicates the effect of DBP, BMI on cardiac parameters and “mediation effect” indicates the effect of coffee consumption on cardiac parameters through DBP, BMI. IVW was used to determine the total effect, direct effect A, and direct effect B; the delta method was used to determine the mediation effect. All statistical tests were two-sided, with significance defined as *P* ≤ 0.05.

### 3.6 Sensitivity analysis

The MR analysis exhibited a consistent direction across the weighted median, MR-Egger, and maximum likelihood methods. However, a degree of heterogeneity was evident in some results (*P* < 0.05). Notwithstanding this, no pleiotropy was detected through the MR-Egger regression, MR-PRESSO (*P* > 0.05). In addition, using funnel plots to analyze all data did not show pleiotropy; the leave-one-out analysis plot failed to confirm the potential SNP-driven causal relationship between exposure and outcome (Figure 3, Supplementary Table S10, Supplementary Figures S9–S23).

### 3.7 Genetic association and direction validation

In the LDSC analysis, coffee consumption is positively genetic associated with several cardiac parameters: LVEDV (Rg = 0.191, *P* < 0.001), LVESV (Rg = 0.144, *P* < 0.001), LVSV (Rg = 0.211, *P* < 0.001), RVEDV (Rg = 0.152, *P* < 0.001), RVESV (Rg = 0.131, *P* = 0.001), and RVSV (Rg = 0.168, *P* < 0.001). As a result of this association, two traits are likely to share a genetic background, in line with what was concluded from the MR analysis (Table 1 and Supplementary Table S3). In addition, the Steiger test results, with all directions being true and a *P*-value less than 0.05, confirm the absence of reverse causality in our findings (Figure 3, Supplementary Tables S4, S20, S21).

### 3.8 Colocalization analysis

The present study identified a causal relationship between coffee consumption and cardiac parameters. For more robust conclusions, we searched public databases for targets of coffee consumption and found that the 7p21 locus near the AHR was the most prominent locus associated with coffee consumption, and it has also been suggested that the AHR may have a key role in the learning or exercise pathways that induce coffee consumption (38, 39). Subsequently, we obtained eQTL signals (*P* < 5 × 10^−8^), referring to AHR, from blood data provided by the eQTL Consortium. eQTL results for AHR (PP.H4 = 98%, SNP = rs4410790) indicated that AHR shares causally variable loci with cardiac structure and function, which also supports the existence of a causal association between coffee consumption and cardiac parameters from another perspective (Supplementary Tables S22, S23).

## 4 Discussion

We utilized LDSC and MR methodologies to probe the genetic association and causal links between coffee consumption and cardiac structure and function. This study leverages innovative statistical techniques to uncover aspects of their relationship. Initially, our LDSC analysis validated a substantial positive genetic association between coffee consumption and cardiac parameters. We also discerned a significant genetic association between coffee consumption and qualifying cardiac metabolic factors, as well as between these factors and cardiac structure and function. Subsequently, our MR analysis revealed a positive association between coffee consumption and both systolic and diastolic cardiac function in an elderly European demographic, corroborating the findings of extensive cohort studies (40, 41). Furthermore, reverse causality was ruled out through the Steiger test. Lastly, via mediation analysis, we discovered that the impact of coffee consumption on cardiac parameters was partially mediated by DBP and BMI, accounting for 4.2%–12.1% and 20.6%–52.8%, respectively. Our findings remained robust across various MR methodologies, each positing different assumptions about horizontal pleiotropy, suggesting it is unlikely to influence the results. Co localization analysis also proves the robustness of the conclusion to a certain extent.

Pathological cardiac remodeling, often a prelude to clinical HF, predominantly stems from imbalances between oxidative stress and antioxidant capabilities within cardiomyocytes, as well as from biomechanical stresses (42). While reactive oxygen species (ROS) play a vital role in preserving redox-dependent pathways, their overproduction can trigger oxidative stress, mediated by inflammatory factors, ultimately culminating in pathological cardiac remodeling (43).

Recent research indicates that the complex effects of coffee on the cardiovascular system are mediated by a multifactorial dynamic balance, which is essentially driven by the dynamic interactions of its bioactive components (such as flavonoids, caffeine, chlorogenic acid, etc.) through dose-dependent and metabolic background sensitivity. Emerging evidence suggests that the flavonoids in coffee coordinate cardioprotection through a tri-mechanism targeting oxidative stress, vascular homeostasis, and fibrotic remodeling. The antioxidant axis involves Adenosine Monophosphate-activated Protein Kinase (AMPK) phosphorylation driving Nuclear factor erythroid 2-related factor 2 (Nrf2) nuclear translocation, directly upregulating the expression of superoxide dismutase and catalase to scavenge ROS, while Akt-mediated Endothelial Nitric Oxide Synthase (eNOS) Ser1177 phosphorylation enhances Nitric Oxide (NO) generation, counteracting Nicotinamide Adenine Dinucleotide Phosphate (NADPH) oxidase activity and indirectly inhibiting ROS production. This synergistic mechanism significantly reduces systemic oxidative damage markers, such as F2-isoprostanes, by 18% (*p* = 0.002) (44, 45). Additionally, vascular homeostasis regulation is achieved through dual pathways: one by directly scavenging superoxide anions to protect NO bioavailability (46), and the other by activating large-conductance calcium-activated potassium channels in vascular smooth muscle, inducing membrane hyperpolarization and reducing calcium influx, thereby collectively reducing vascular resistance (47, 48). Crucially, the antifibrotic effects exhibit a hierarchical regulatory feature: cGMP/PKG pathway blockade of Smad2/3 nuclear translocation provides direct signal inhibition, while the epigenetic remodeling induced by dual inhibition of DNMT3a/HDAC5 offers sustained transcriptional suppression (49, 50). Furthermore, low concentrations of caffeine mitigate oxidative stress through multi-target mechanisms, including adenosine A1 receptor antagonism, cAMP elevation, and suppression of pro-inflammatory mediators such as Interleukin-6 (IL-6) (51). Conversely, elevated caffeine concentrations may intensify oxidative stress by further antagonizing A2 receptors (52).

Previous studies have reported that over 95% of caffeine is metabolized by cytochrome P450 1A2 (CYP1A2). Research has confirmed that a common polymorphism, rs762551, in the CYP1A2 gene can alter caffeine metabolism by decreasing enzyme activity and inducibility (53). Specifically, individuals with the AC and CC genotypes of this polymorphism are commonly referred to as “slow metabolizers”, while individuals with the AA genotype are defined as “fast metabolizers”. Slow metabolizers may have reduced CYP1A2 enzyme activity, leading to slower caffeine breakdown in the body. On the other hand, fast metabolizers with the AA genotype have more efficient CYP1A2 enzymes, resulting in faster caffeine metabolism (53). Notably, studies have shown that CYP1A2 levels in males appear to be higher than in females (54–56), which may be related to smoking, as CYP1A2 expression is easily induced by smoking (57). Estrogen, on the other hand, can significantly reduce CYP1A2 levels, which is part of the reason why male CYP1A2 levels are higher than those in females, as confirmed by previous studies (58). Pregnancy or exogenous estrogen supplementation can further lower its activity and inhibit caffeine metabolism (59, 60). Research stratified by CYP1A2 genotype has shown significant differences between the two groups: slow metabolizers (AC/CC genotype) begin to show an increased risk of myocardial infarction when consuming more than 2 cups daily (OR = 1.64, 95% CI 1.14–2.34), while fast metabolizers (AA genotype) do not show an increased risk of myocardial infarction even with high intake, and a trend toward a decreased risk of myocardial infarction was observed, though the difference was not statistically significant (61). Additionally, a multicenter cohort study showed that among slow metabolizers, coffee consumption also increased the risk of impaired fasting glucose in hypertensive patients (62). Thus, gender-related differences in the effects of coffee intake may be partially attributed to gender-specific enzyme expression regulation (such as estrogen inhibition and smoking induction).

Chlorogenic acids (CGAs) play a crucial role in preventing free radical damage, inhibiting inflammatory factors, improving insulin resistance, enhancing endothelial NO production (63). Prolonged pulmonary arterial hypertension manifests a close association with the pathological remodeling of the RV (64). Research suggests that CGAs can efficaciously ameliorate pulmonary arterial hypertension, primarily by directly inhibiting c-Src tyrosine kinase activity and reducing ROS levels, consequently improving pathological proliferation of pulmonary arterial smooth muscle cells (65). Coffee contains melanoidins and diterpenoids, also substances known for their antioxidant properties. However, the roasting process, while yielding these beneficial compounds, can also produce harmful substances. One such substance is acrylamide, noted for its neurotoxicity and potential carcinogenic activity. Encouragingly, advancements in the roasting process may mitigate this harm (40).

Despite the abundance of studies on the cardiovascular implications of coffee consumption, investigations into its influence on the heart's structure and functionality remain limited. Parameters from cardiac MRI, like LVSV, RVSV, LVESV, RVESV, LVEF, and RVEF, serve as indicators of ventricular systolic function, with LVEDV, and RVEDV acting as a marker for ventricular compliance during diastole and myocardial remodeling (63). Expectedly, the heart's structure and function decline with age, such as LVEDV, LVESV, and LVSV (41, 66); however, our findings reveal a contrasting trend. This could be attributed to the potential neutralizing effect on oxidative stress (67). We posit that such benefits might be more pronounced in populations enduring chronic oxidative stress, such as individuals with hypertension and diabetes. Regrettably, the constraints of GWAS data prevent an in-depth exploration of coffee's effects on the health of the elderly population. Experimental studies have demonstrated that supplementing mice with coffee extract can ameliorate LV dilation, alleviate myocardial fibrosis, and enhance ventricular contractile function (68). Two large-scale epidemiological studies evaluate the influence of coffee consumption on ventricular structure and function. The findings consistently indicate a significant association between coffee consumption and improved cardiac function. Subgroup analysis reveals an association between consuming more than 4 cups of coffee daily and compromised LV function, in line with previous research (8, 40). Based on genetic studies, coffee consumption has an impact on cardiovascular disease, and the effects of coffee primarily originate from its complex bioactive activity (69). Factors such as the coffee type, consumption quantity, and additional ingredients could potentially confound the outcomes of such studies. Moreover, the constraints inherent in GWAS data cannot determine whether there is a U-shaped relationship.

Our two-step MR analysis identified DBP reduction and BMI elevation as potential mediators linking coffee consumption to cardiac structural remodeling. However, it necessitates careful reconciliation with existing evidence on coffee's cardiometabolic impacts. The observed DBP-lowering effect contrasts with acute caffeine-induced vasoconstriction but aligns with long-term vascular adaptations. Chronic coffee consumption (≥3 cups/day) has been associated with improved endothelial function through polyphenol-mediated nitric oxide bioavailability, particularly chlorogenic acid, which reduces arterial stiffness in hypertensive populations (70, 71). A large-scale study comprising 9,009 participants demonstrated that linear regression models, adjusted for age, sex, BMI, diabetes, hypertension, smoking status, and additive use, indicated a significant inverse relationship between moderate and high coffee consumption and reductions in both SBP and DBP (72). This may reflect compensatory mechanisms where adenosine receptor antagonism from caffeine is counterbalanced by antioxidant-mediated vasodilation over time. A multitude of studies posit that CGAs effectively manages weight and abdominal obesity in obese individuals (73, 74). The majority of prior studies have indicated an inverse relationship between coffee consumption and BMI (75). Nevertheless, a Korean study highlights that the consumption of coffee containing added sugar or cream may be associated with an elevated risk of obesity (76). Recently, a large-scale prospective cohort study conducted in the United States thoroughly investigated the associations between coffee consumption, added sugar, and long-term weight changes. The findings revealed that each additional daily cup of unsweetened caffeinated coffee and unsweetened decaffeinated coffee was associated with a 0.12 kg reduction in body weight over a 4-year period. In contrast, the addition of one teaspoon of sugar per day was associated with a 0.09 kg weight gain over the same duration. Notably, no significant association was observed between the habitual use of cream or non-dairy coffee whitener and changes in body weight (77). The positive mediation role of BMI identified in our study is also inconsistent with evidence from experimental studies indicating that caffeine-induced thermogenesis and appetite suppression contribute to the amelioration of obesity (78). However, our genetic instruments likely capture “real-world” coffee consumption patterns, including caloric additives (e.g., sugar, cream) prevalent in commercial coffee products. Future research should stratify participants by coffee type (espresso vs. filtered) and the use of additives to better understand the differential impacts of these factors on health outcomes.

The detrimental effects of hypertension and obesity on cardiac architecture and functionality are well documented. Chronic hypertension can induce micro- and macro-alterations in the myocardium, encompassing cardiac fibrosis, ventricular and arterial system remodeling, and adaptive modifications in cardiac functionality (79). A cohort study discovered a positive association between cumulative BMI, hypertension burden, and LV hypertrophy during middle age. Furthermore, irrespective of the focus being on the left or right heart, obesity can impinge on its systolic and diastolic functionalities (80, 81). Studies have demonstrated that post-bariatric surgery weight reduction can ameliorate subclinical cardiac functionality and reverse ventricular remodeling (82). The influence of obesity on the heart is multifaceted, possibly attributed to an escalation in total circulating blood volume and cardiac pressure load, resulting in long-term adaptive heart enlargement and dilation and changes in cardiac functional adaptability, or via mechanisms such as inflammation, hormonal shifts, metabolic disturbances, and microvascular diseases (81, 83).

This study presents numerous noteworthy advantages. Firstly, the GWAS data, derived from a substantial European population consortium, enhances the analysis's statistical power. Secondly, the association between coffee consumption and the structure and functionality of the heart is elucidated from a genetic standpoint via LDSC, MR, and colocalization analysis. Thirdly, given that genetic variations are inherent, natural, and randomly assigned at conception, MR analysis offers dependable estimates. Lastly, the implementation of a two-step MR analysis helps mitigate biases arising from confounding among exposure, mediator, and outcome.

Nonetheless, the study also displays certain limitations. Primarily, the presence of heterogeneity in some results could introduce biases. However, we lacked access to GWAS data from varied age, gender, and health status groups to examine the sources of this heterogeneity. Secondly, the data suggests that coffee consumption correlates positively with BMI, serving as a contradiction to previous studies that associated coffee with obesity. This discrepancy may be attributed to the questionnaire's limitations in providing detailed information about coffee consumption, appetite post-coffee intake, and daily medication usage. Thirdly, while higher coffee consumption correlates with improved cardiac structure and function, it cannot be definitively concluded that the observed increase in cardiac parameters is not influenced by obesity-related increases in blood volume and adaptive cardiac enlargement.

## 5 Conclusion

This study confirms through MR analysis that coffee consumption in the elderly European population is genetically associated with improvements in cardiac systolic/diastolic function (mediating proportions: DBP 4.2%–12.1%, BMI 20.6%–52.8%), suggesting that enhanced management of blood pressure and obesity may improve cardiovascular health. Future research should focus on exploring the dynamic interactions between coffee's bioactive components (such as flavonoids and chlorogenic acid) and metabolic factors in populations experiencing chronic oxidative stress, and differentiate the heterogeneous effects of coffee types and additives on cardiac structure and function.

## Data Availability

The original contributions presented in the study are included in the article/Supplementary Material, further inquiries can be directed to the corresponding author.
