# Supplementary material for: Evaluating the effects of coffee consumption on the structure and function of the heart from multiple perspectives
Source: Front Cardiovasc Med. 2025 Apr 15;12:1453106. doi: 10.3389/fcvm.2025.1453106 (PMC12037506; doi:10.3389/fcvm.2025.1453106)
Supplement: Supplementary file 1 [file Datasheet1.pdf]

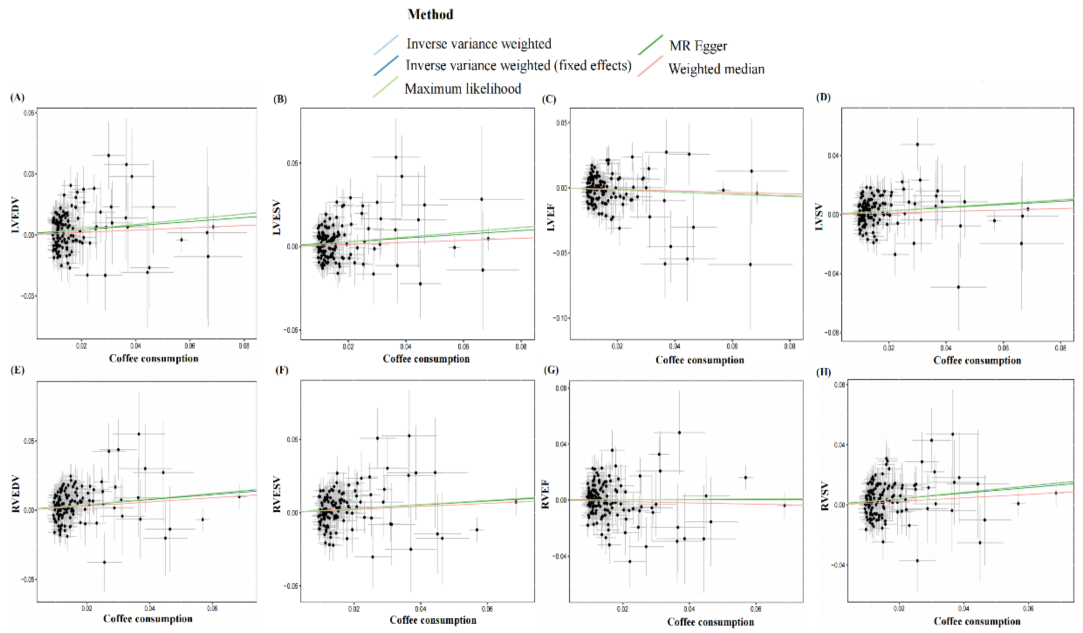

Supplementary Figure 1. Scatterplots of SNP effects on coffee consumption versus their effects on LVEDV (A), LVESV (B), LVEF (C), LVSV (D), RVEDV (E), RVESV (F), RVEF (G), and RVSV (H) with the slope of each line corresponding to the estimated MR effect of inverse variance-weighted (re), inverse variance-weighted (fe), weighted median, MR-Egger, and maximum likelihood, respectively. Error bars indicate 95% CIs. SNPs, single nucleotide polymorphisms; LVEDV, Left ventricular end-diastolic volume; LVESV, Left ventricular end-systolic volume; LVEF, Left ventricular ejection fraction; LVSV, Left ventricle stroke volume; RVEDV, Right ventricular end-diastolic volume; RVESV, Right ventricular end-systolic volume; RVEF, Right ventricular ejection fraction; RVSV, Right ventricle stroke volume.

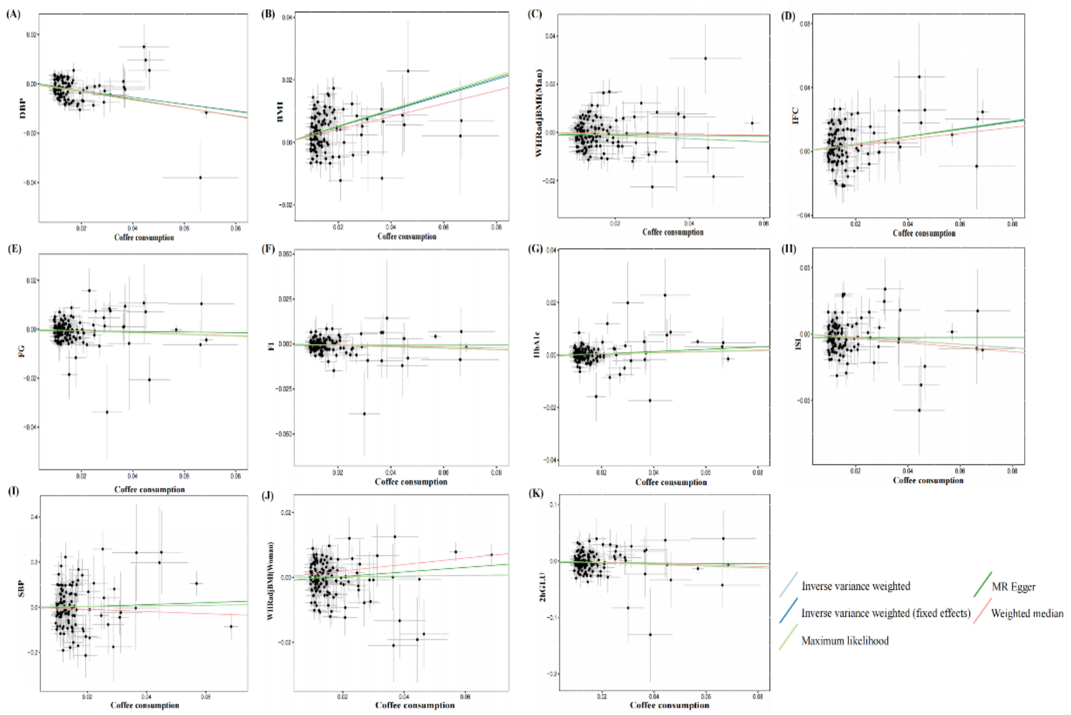

Supplementary Figure 2. Scatterplots of SNP effects on coffee consumption versus their effects on DBP (A), BMI (B), WHRadjBMI(Man) (C), IFC (D), FG (E), FI (F), HbA1c (G), ISI (H), SBP (I), WHRadjBMI(Man) (J), and 2hGLU (K) with the slope of each line corresponding to the estimated MR effect of inverse variance-weighted (re), inverse variance-weighted (fe), weighted median, MR-Egger, and maximum likelihood, respectively. Error bars indicate 95% CIs. SNPs, single nucleotide polymorphisms; DBP, Diastolic blood pressure; SBP, Systolic blood pressure; BMI, Body mass index; WHRadjBMI, Waist-to-hip ratio adjusted for body mass index; FI, Fasting insulin; FG, Fasting glucose; 2hGLU, 2h-glucose post-challenge; HbA1c, glycated hemoglobin; ISI, Modified Stumvoll Insulin Sensitivity Index; IFC, Insulin Fold Change.

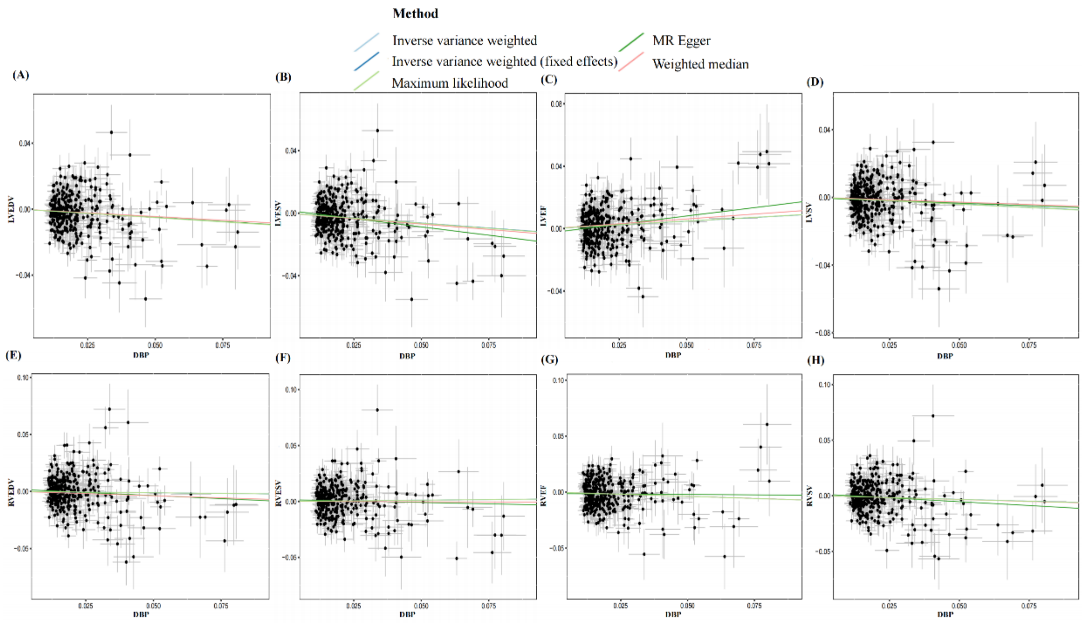

Supplementary Figure 3. Scatterplots of SNP effects on DBP versus their effects on LVEDV (A), LVESV (B), LVEF (C), LVSV (D), RVEDV (E), RVESV (F), RVEF (G), and RVSV (H) with the slope of each line corresponding to the estimated MR effect of inverse variance-weighted (re), inverse variance-weighted (fe), weighted median, MR-Egger, and maximum likelihood, respectively. Error bars indicate 95% CIs. SNPs, single nucleotide polymorphisms; DBP, Diastolic blood pressure. The explanation of other abbreviations is the same as Supplementary Figure 1.

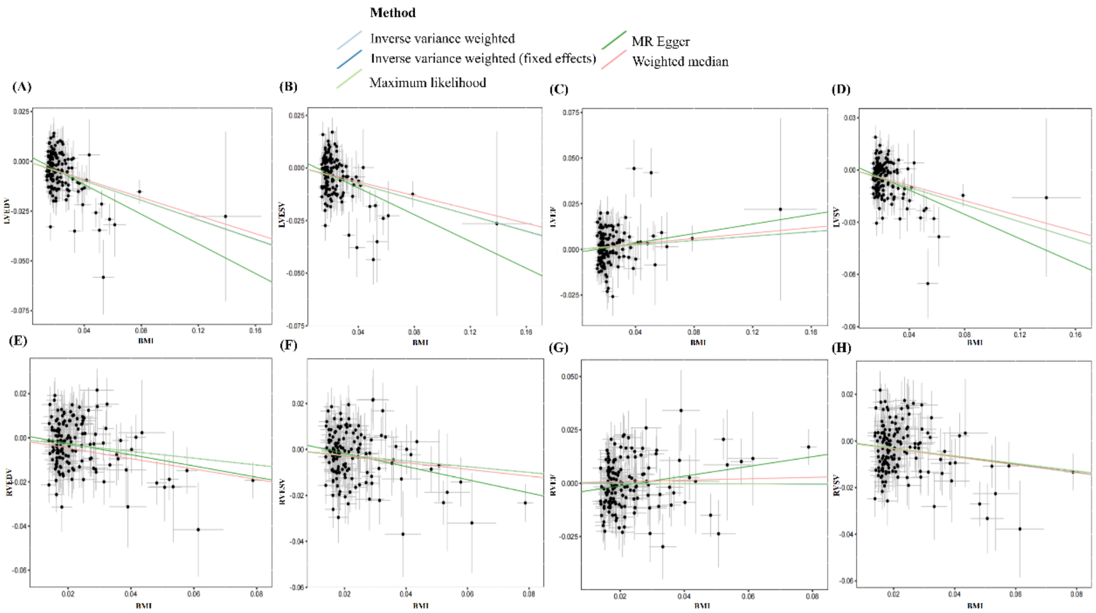

Supplementary Figure 4. Scatterplots of SNP effects on BMI versus their effects on LVEDV (A), LVESV (B), LVEF (C), LVSV (D), RVEDV (E), RVESV (F), RVEF (G), and RVSV (H) with the slope of each line corresponding to the estimated MR effect of inverse variance-weighted (re), inverse variance-weighted (fe), weighted median, MR-Egger, and maximum likelihood, respectively. Error bars indicate 95% CIs. BMI, Body mass index. The explanation of other abbreviations is the same as Supplementary Figure 1.

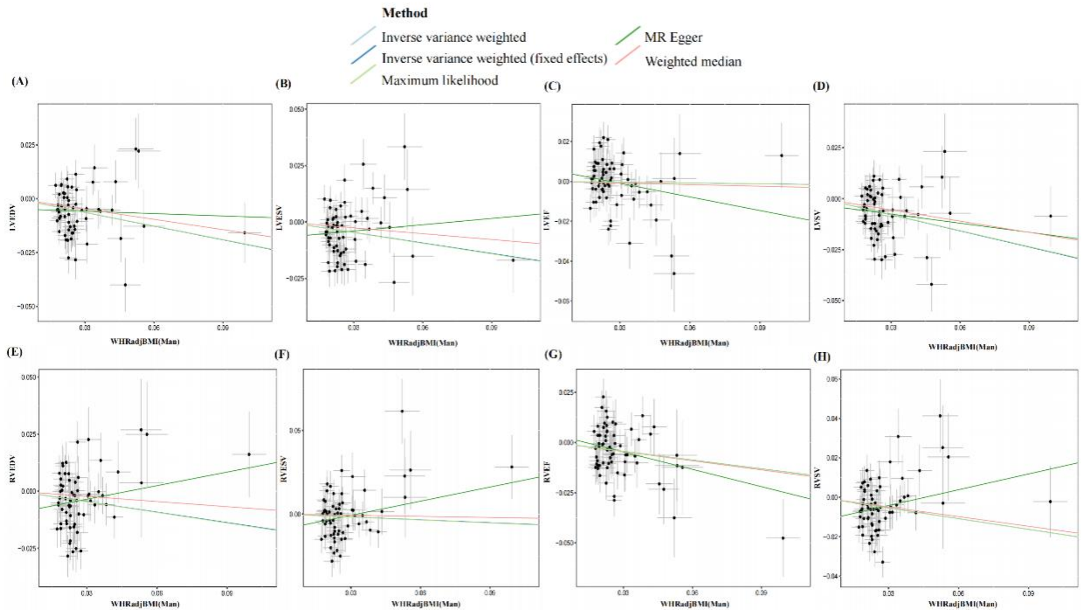

Supplementary Figure 5. Scatterplots of SNP effects on WHRadjBMI(Man) versus their effects on LVEDV (A), LVESV (B), LVEF (C), LVSV (D), RVEDV (E), RVESV (F), RVEF (G), and RVSV (H) with the slope of each line corresponding to the estimated MR effect of inverse variance-weighted (re), inverse variance-weighted (fe), weighted median, MR-Egger, and maximum likelihood, respectively. Error bars indicate 95% CIs. WHRadjBMI, Waist-to-hip ratio adjusted for body mass index. The explanation of other abbreviations is the same as Supplementary Figure 1.

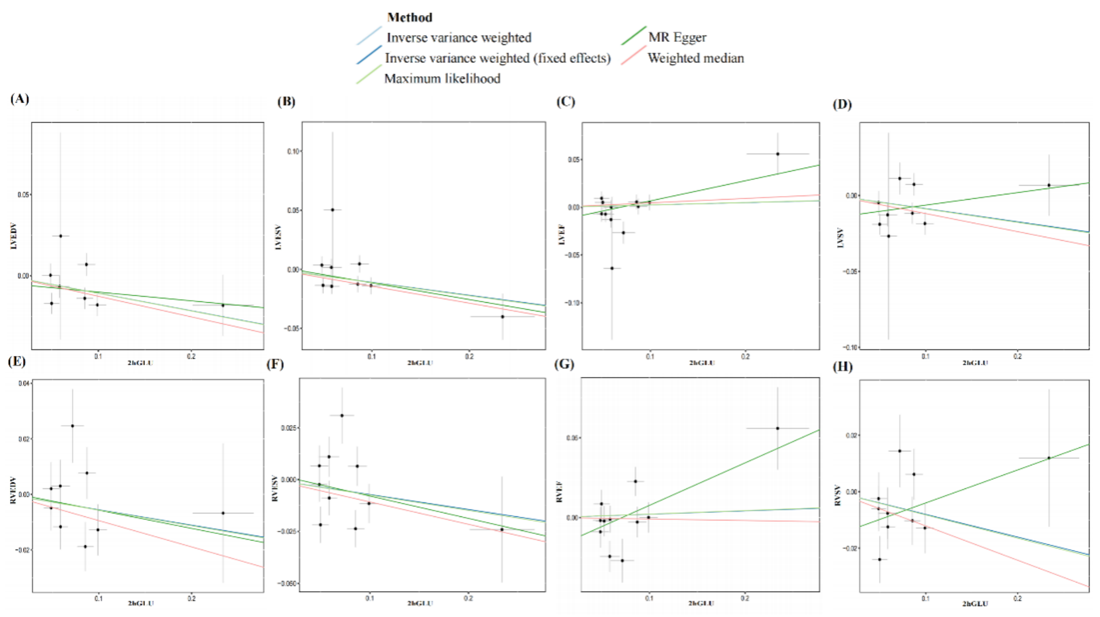

Supplementary Figure 6. Scatterplots of SNP effects on 2hGLU versus their effects on LVEDV (A), LVESV (B), LVEF (C), LVSV (D), RVEDV (E), RVESV (F), RVEF (G), and RVSV (H) with the slope of each line corresponding to the estimated MR effect of inverse variance-weighted (re), inverse variance-weighted (fe), weighted median, MR-Egger, and maximum likelihood, respectively. Error bars indicate 95% CIs. 2hGLU, 2h-glucose post-challenge. The explanation of other abbreviations is the same as Supplementary Figure 1.

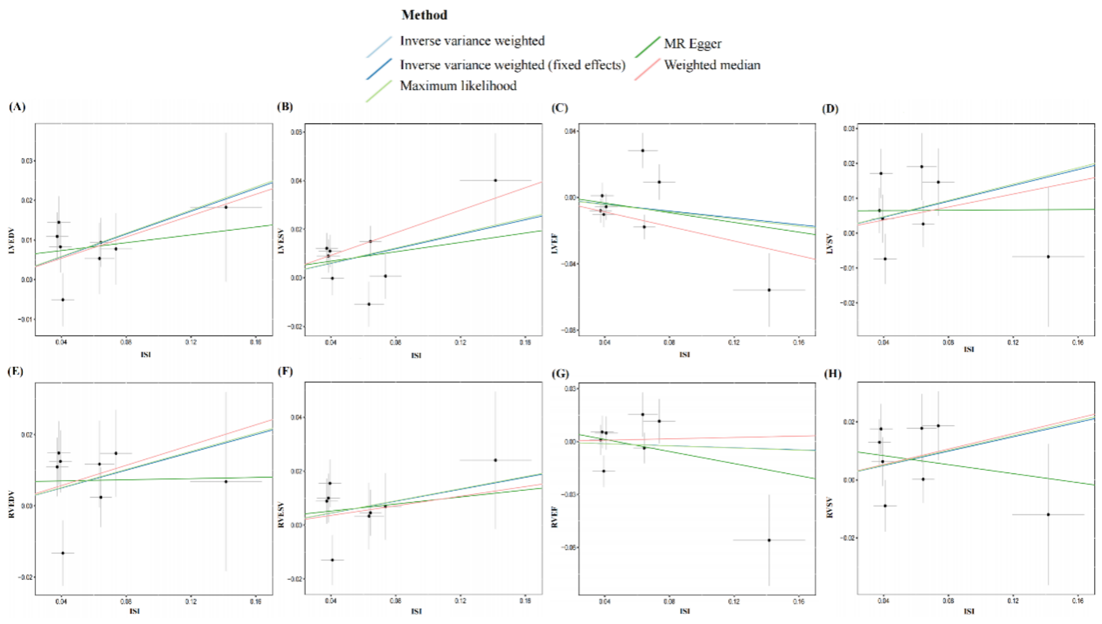

Supplementary Figure 7. Scatterplots of SNP effects on ISI versus their effects on LVEDV (A), LVESV (B), LVEF (C), LVSV (D), RVEDV (E), RVESV (F), RVEF (G), and RVSV (H) with the slope of each line corresponding to the estimated MR effect of inverse variance-weighted (re), inverse variance-weighted (fe), weighted median, MR-Egger, and maximum likelihood, respectively. Error bars indicate 95% CIs. ISI, Modified Stumvoll Insulin Sensitivity Index. The explanation of other abbreviations is the same as Supplementary Figure 1.

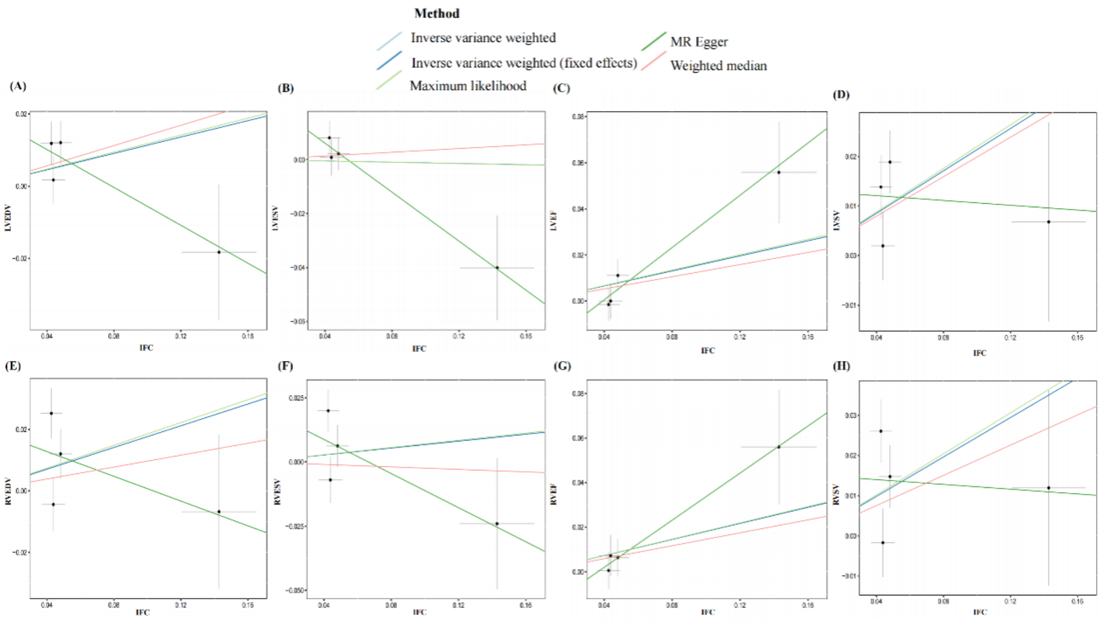

Supplementary Figure 8. Scatterplots of SNP effects on IFC versus their effects on LVEDV (A), LVESV (B), LVEF (C), LVSV (D), RVEDV (E), RVESV (F), RVEF (G), and RVSV (H) with the slope of each line corresponding to the estimated MR effect of inverse variance-weighted (re), inverse variance-weighted (fe), weighted median, MR-Egger, and maximum likelihood, respectively. Error bars indicate 95% CIs. IFC, Insulin Fold Change. The explanation of other abbreviations is the same as Supplementary Figure 1.

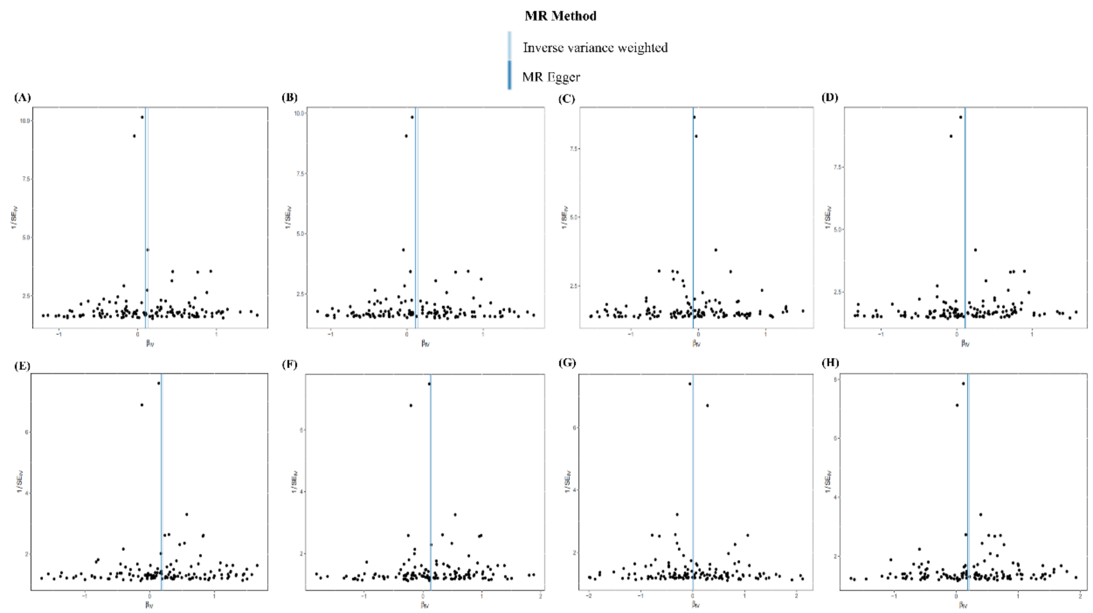

Supplementary Figure 9. Funnel plots of the association between Coffee comsumption and LVEDV(A), LVESV(B), LVEF(C), LVSV(D), RVEDV(E), RVESV(F), RVEF(G), RVSV(H). The explanation of other abbreviations is the same as Supplementary Figure 1.

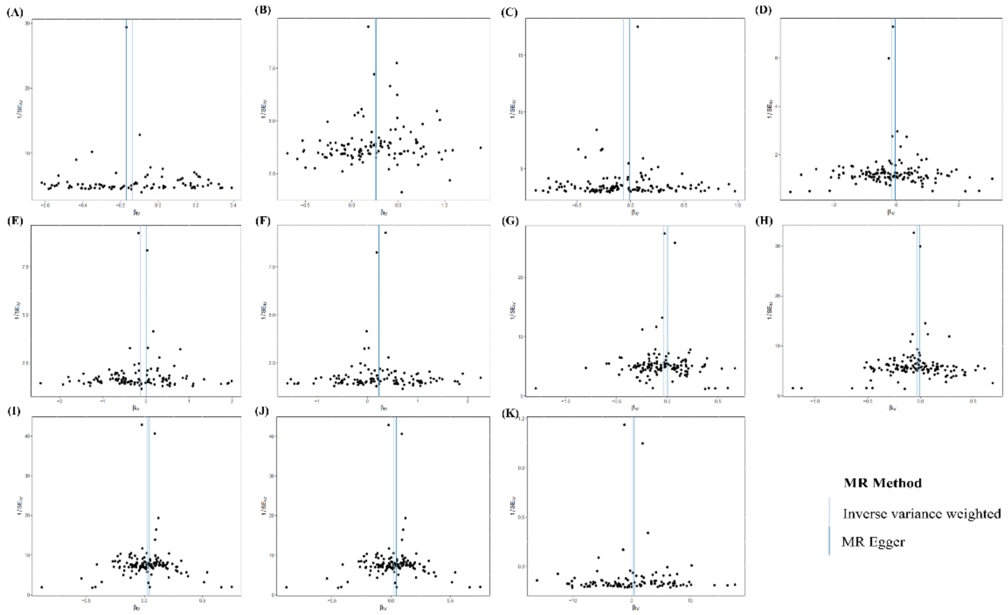

Supplementary Figure 10. Funnel plots of the association between Coffee comsumption and DBP(A), BMI(B), WHRadjBMI(Man)(C), 2hGLU(D), ISIadjBMI(E), IFCadjBMI(F), FI(G), FG(H), HbA1c(I), WHRadjBMI(Woman)(J), SBP(K). The explanation of other abbreviations is the same as Supplementary Figure 2.

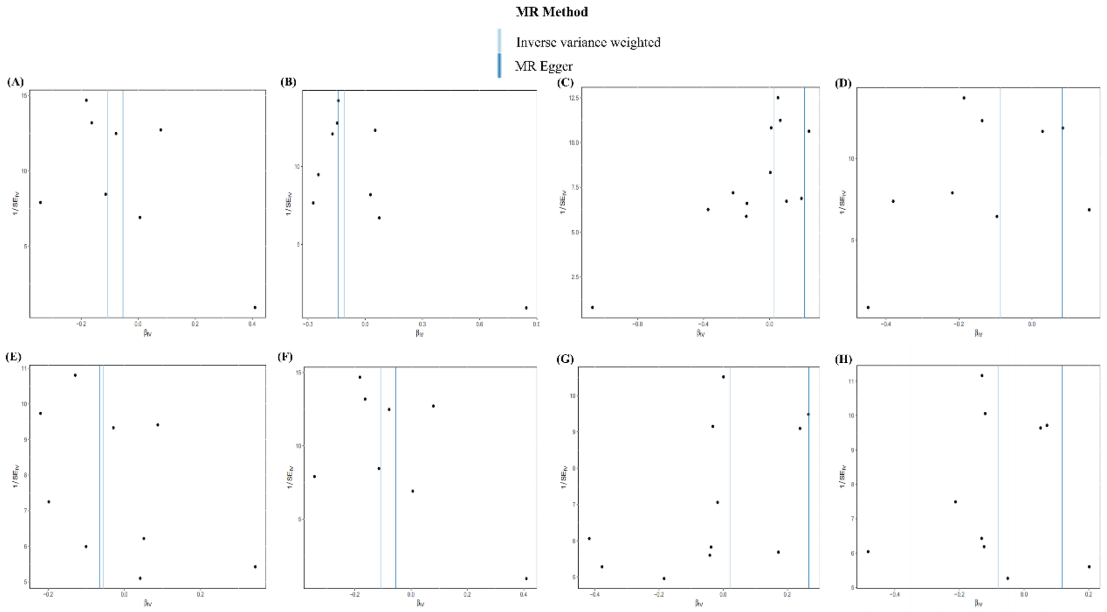

Supplementary Figure 11. Funnel plots of the association between 2hGLU and LVEDV (A), LVESV (B), LVEF (C), LVSV (D), RVEDV (E), RVESV (F), RVEF (G), and RVSV (H). The explanation of other abbreviations is the same as Supplementary Figure 1.

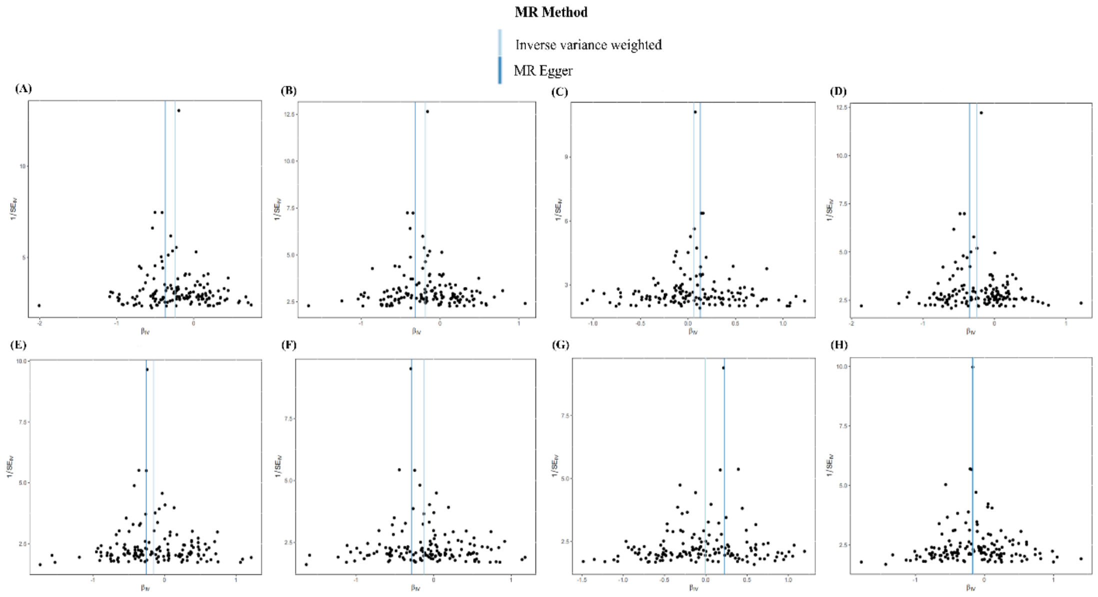

Supplementary Figure 12. Funnel plots of the association between BMI and LVEDV (A), LVESV (B), LVEF (C), LVSV (D), RVEDV (E), RVESV (F), RVEF (G), and RVSV (H). The explanation of other abbreviations is the same as Supplementary Figure 1.

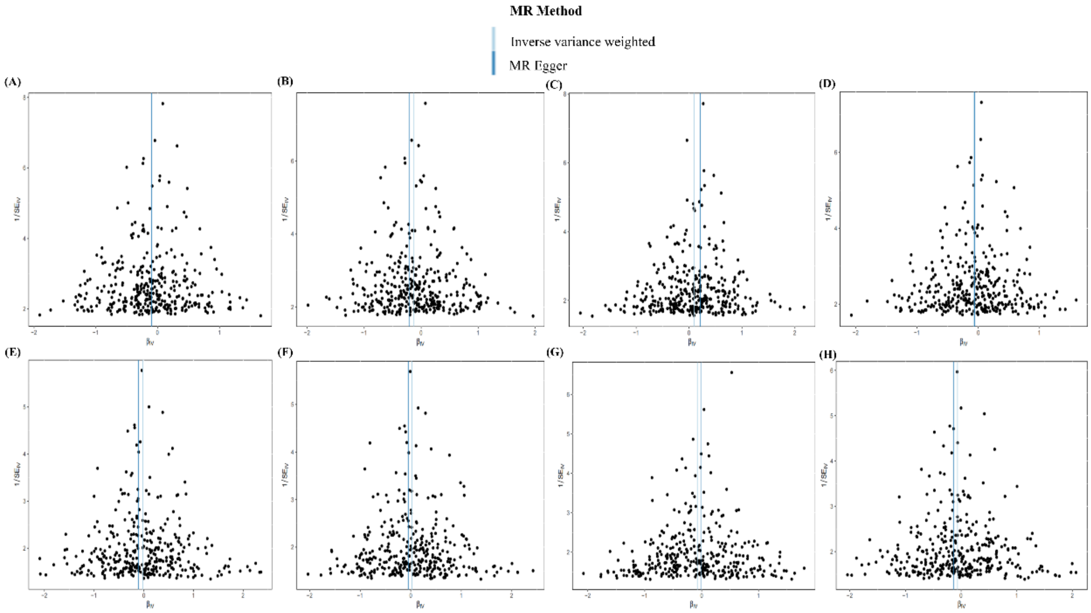

Supplementary Figure 13. Funnel plots of the association between DBP and LVEDV (A), LVESV (B), LVEF (C), LVSV (D), RVEDV (E), RVESV (F), RVEF (G), and RVSV (H). The explanation of other abbreviations is the same as Supplementary Figure 1.

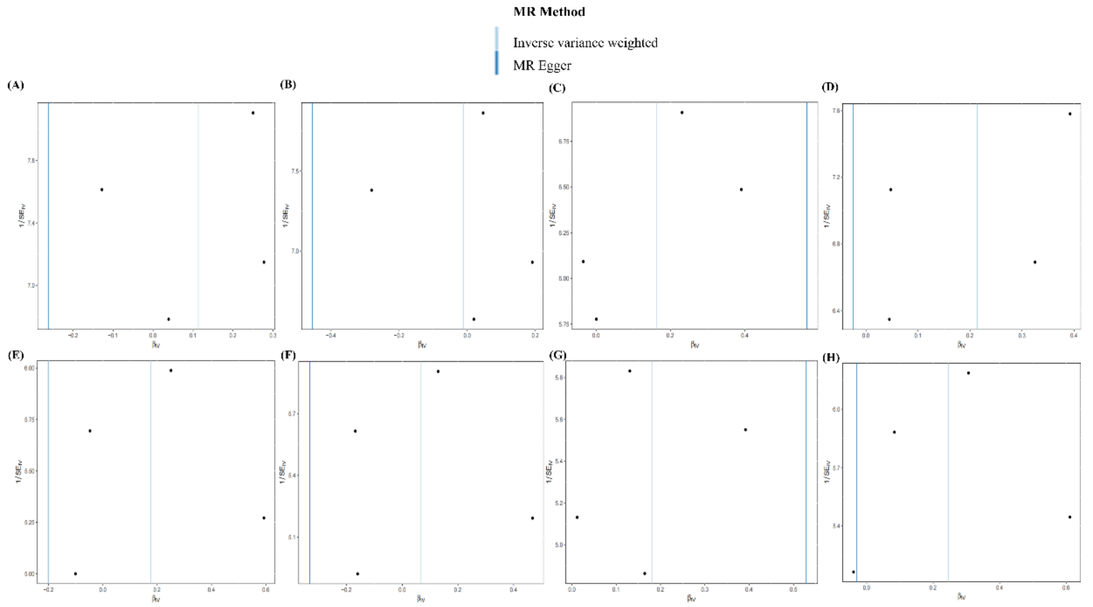

Supplementary Figure 13. Funnel plots of the association between IFC and LVEDV (A), LVESV (B), LVEF (C), LVSV (D), RVEDV (E), RVESV (F), RVEF (G), and RVSV (H). The explanation of other abbreviations is the same as Supplementary Figure 1.

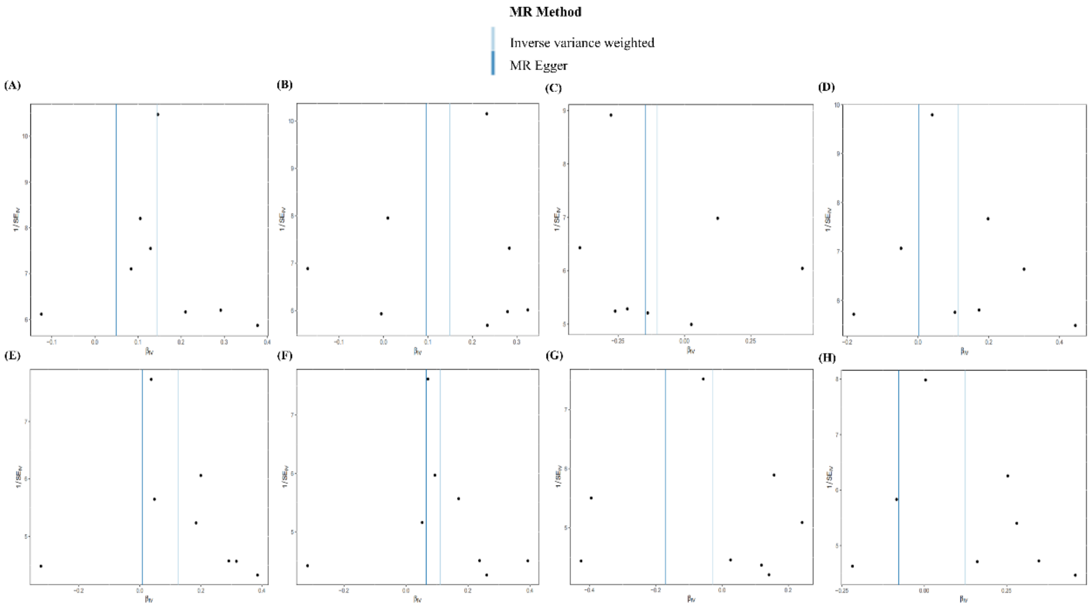

Supplementary Figure 14. Funnel plots of the association between ISI and LVEDV (A), LVESV (B), LVEF (C), LVSV (D), RVEDV (E), RVESV (F), RVEF (G), and RVSV (H). The explanation of other abbreviations is the same as Supplementary Figure 1.

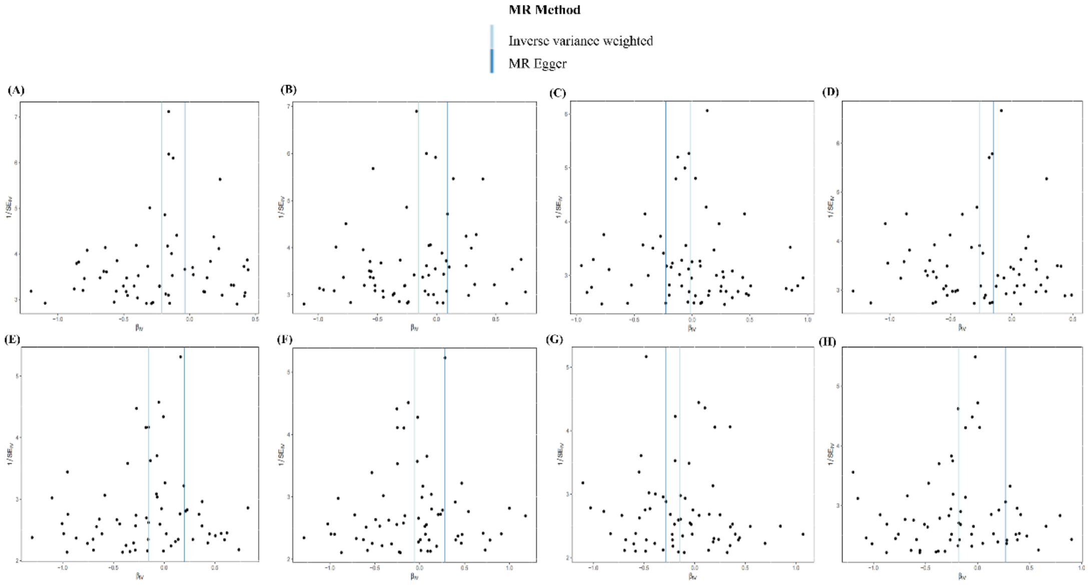

Supplementary Figure 15. Funnel plots of the association between WHRadjBMI(Man) and LVEDV (A), LVESV (B), LVEF (C), LVSV (D), RVEDV (E), RVESV (F), RVEF (G), and RVSV (H). The explanation of other abbreviations is the same as Supplementary Figure 1.

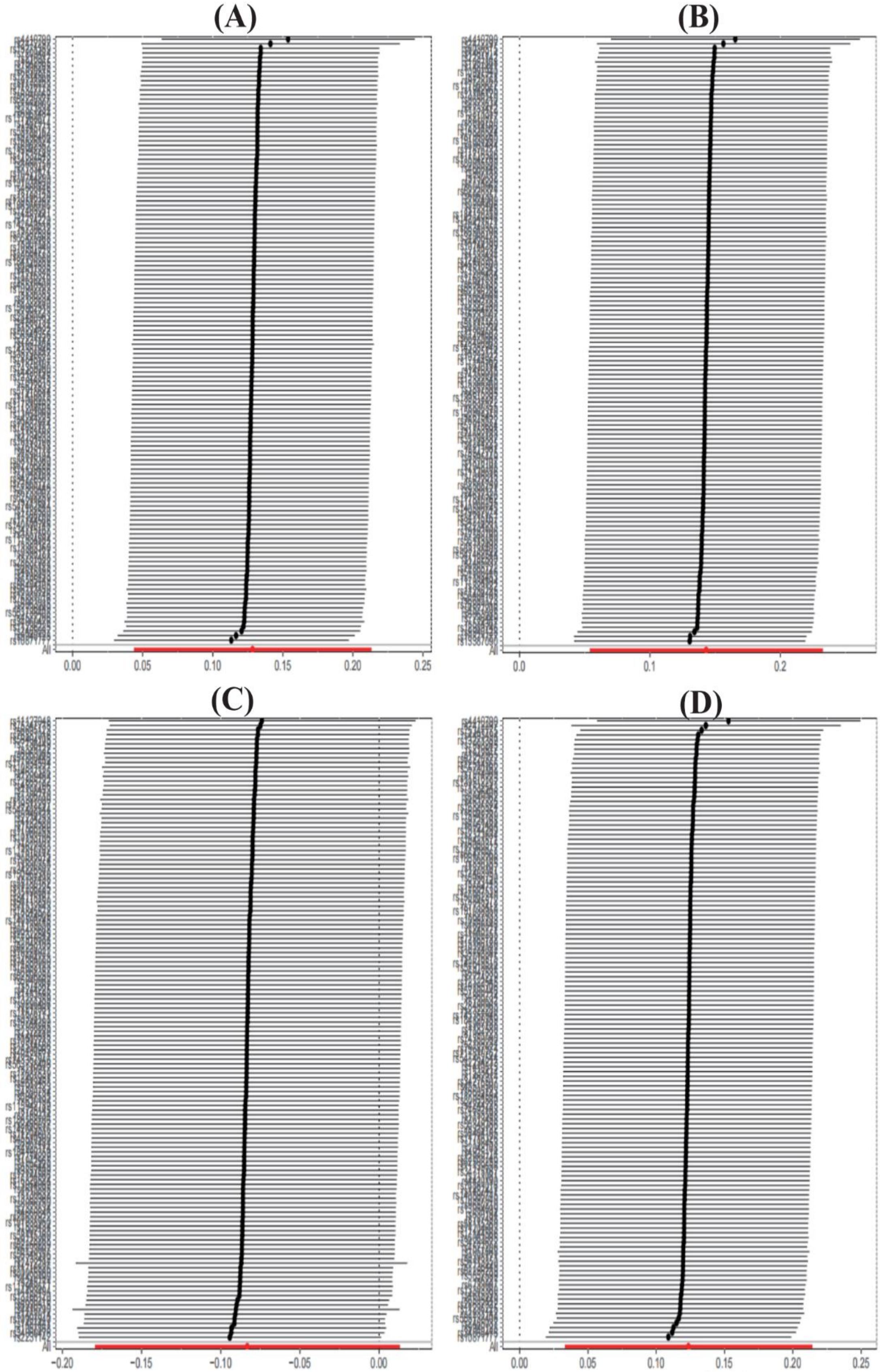

MR leave-one-out sensitivity analysis for ‘exposure’on ‘outcome’

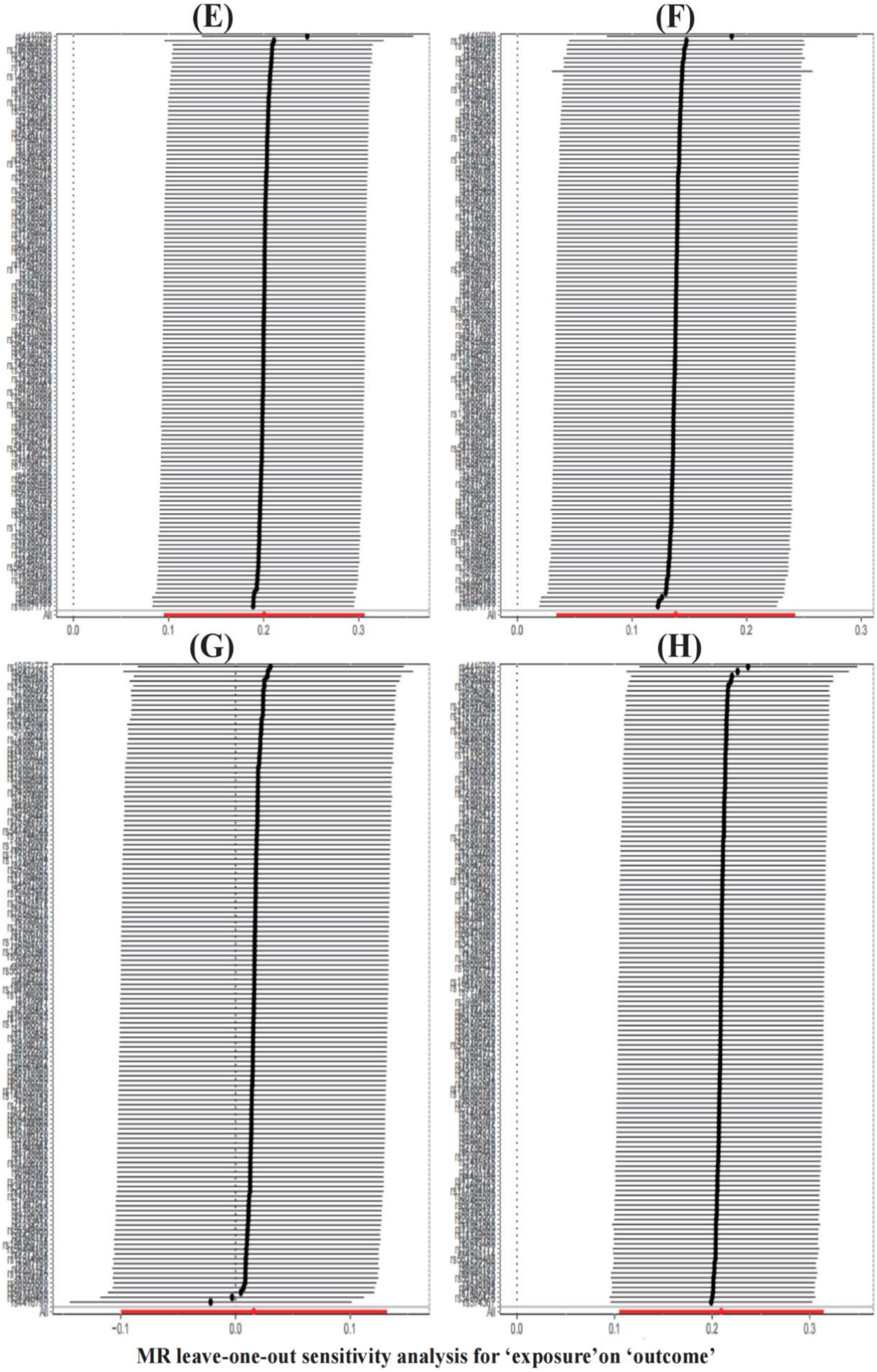

Supplementary Figure 16. Leave-one-out analyses of the association between Coffee comsumption and LVEDV (A), LVESV (B), LVEF (C), LVSV (D), RVEDV (E), RVESV (F), RVEF (G), RVSV (H).

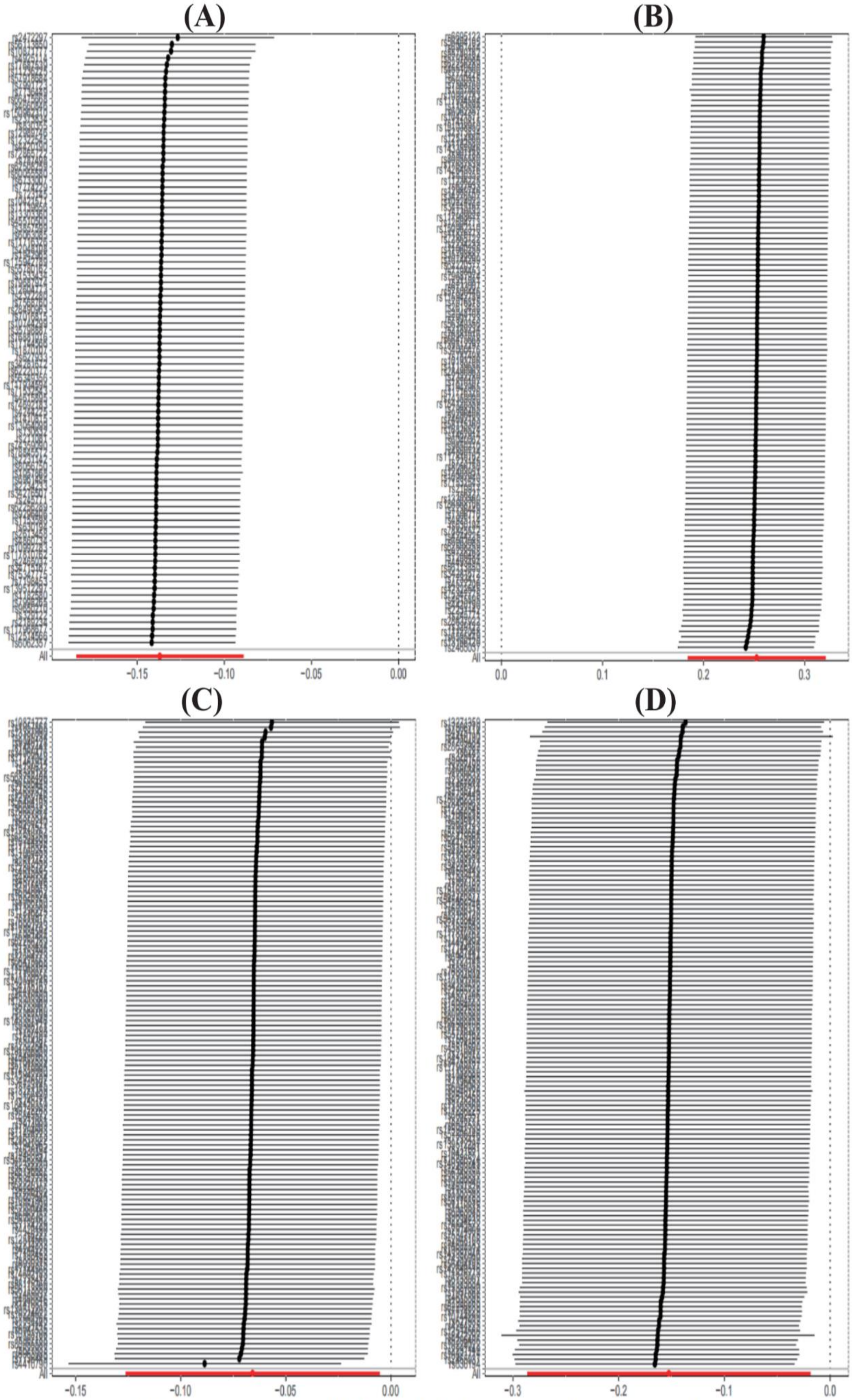

MR leave-one-out sensitivity analysis for 'exposure'on 'outcome'

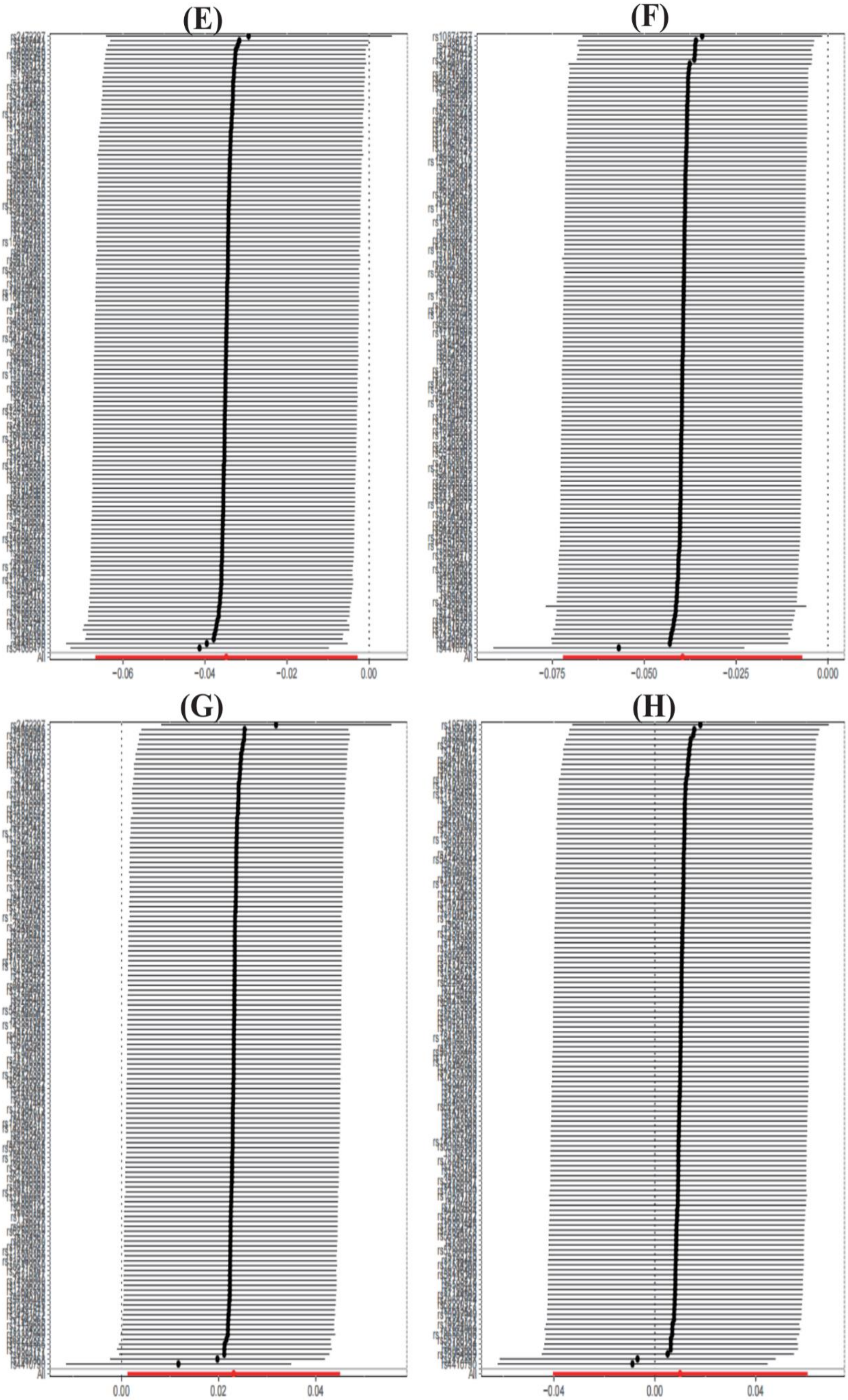

MR leave-one-out sensitivity analysis for 'exposure'on 'outcome'

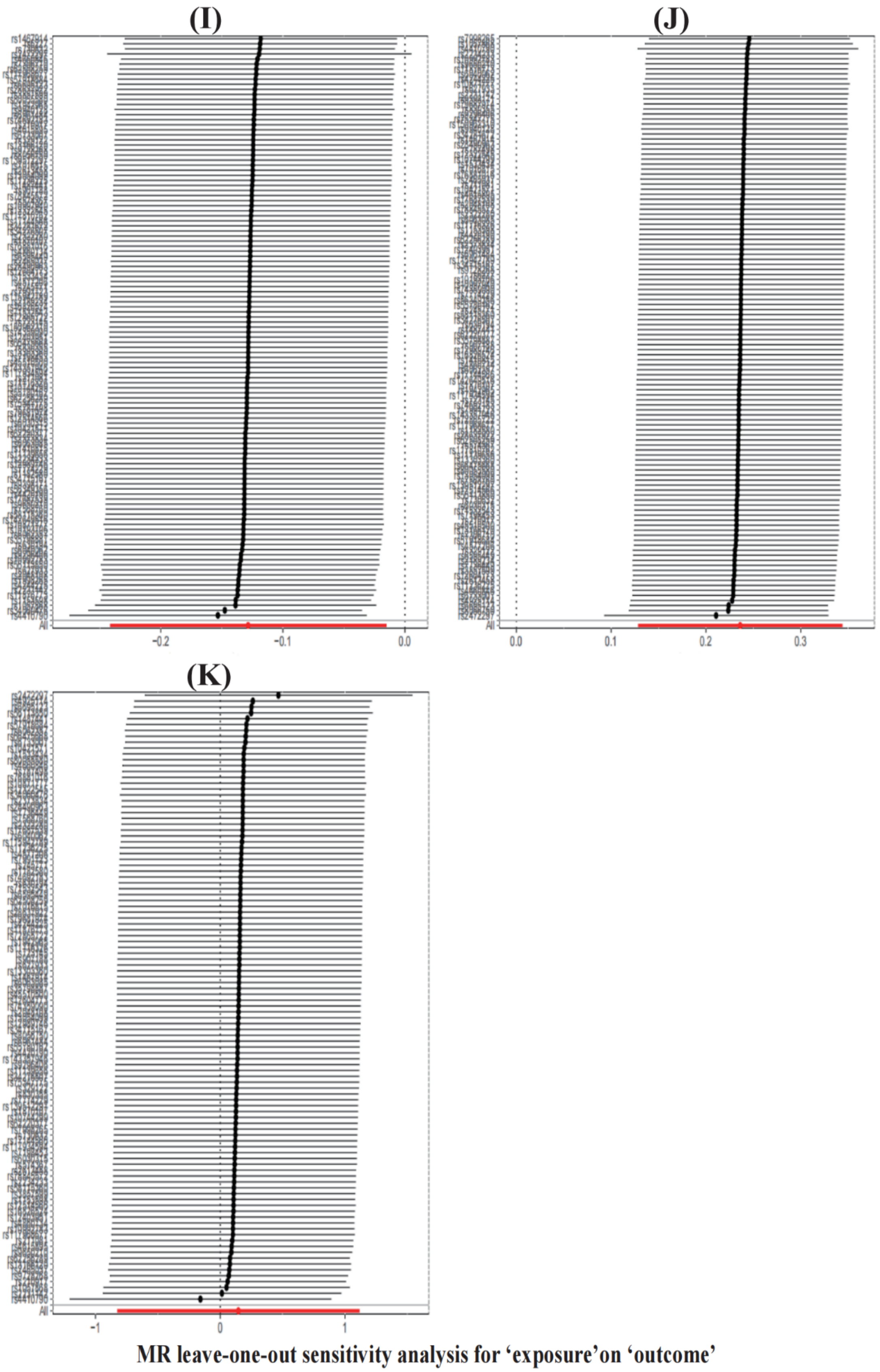

Supplementary Figure 17. Leave-one-out analyses of the association between Coffee comsumption and DBP (A), BMI (B), WHRadjBMI(Man) (C), 2Hglu (D), FG (E), FI (F), HbA1c (G), WHRadjBMI (Woman)((H), ISIadjBMI (I), IFCadjBMI (J), SBP (K).

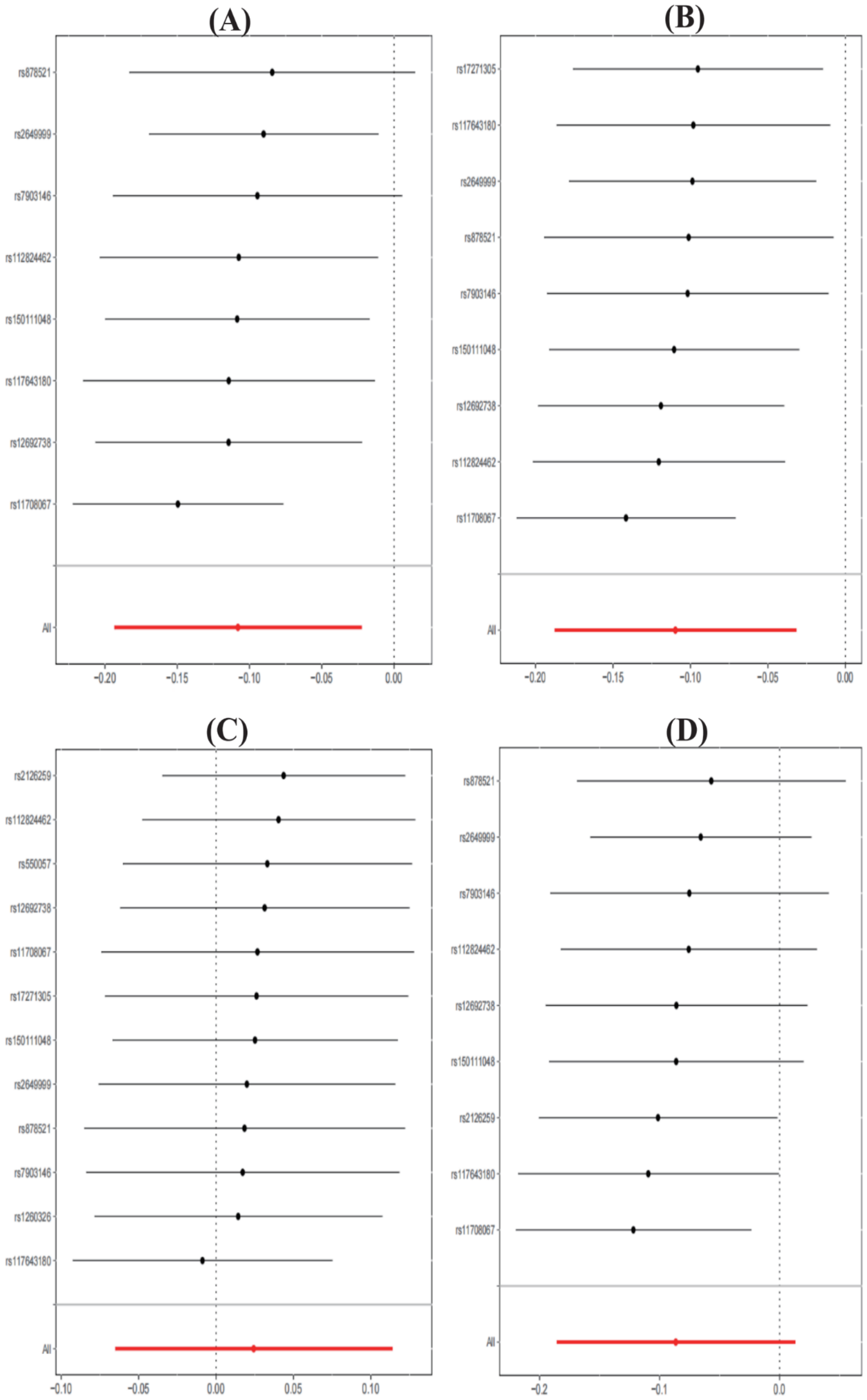

MR leave-one-out sensitivity analysis for 'exposure'on 'outcome'

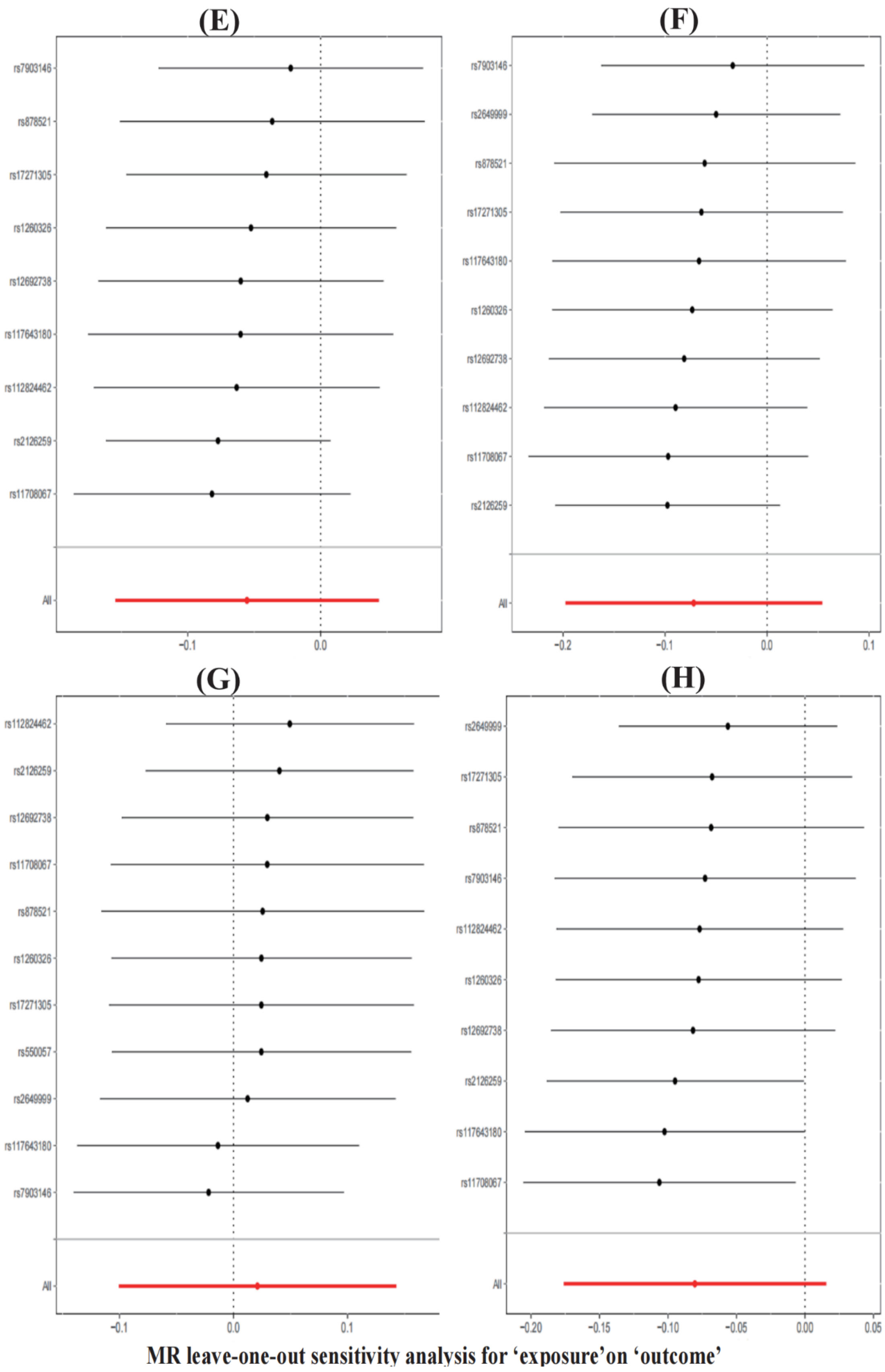

Supplementary Figure 18. Leave-one-out analyses of the association between 2hGLU and LVEDV (A), LVESV (B), LVEF (C), LVSV (D), RVEDV (E), RVESV (F), RVEF (G), RVSV (H).

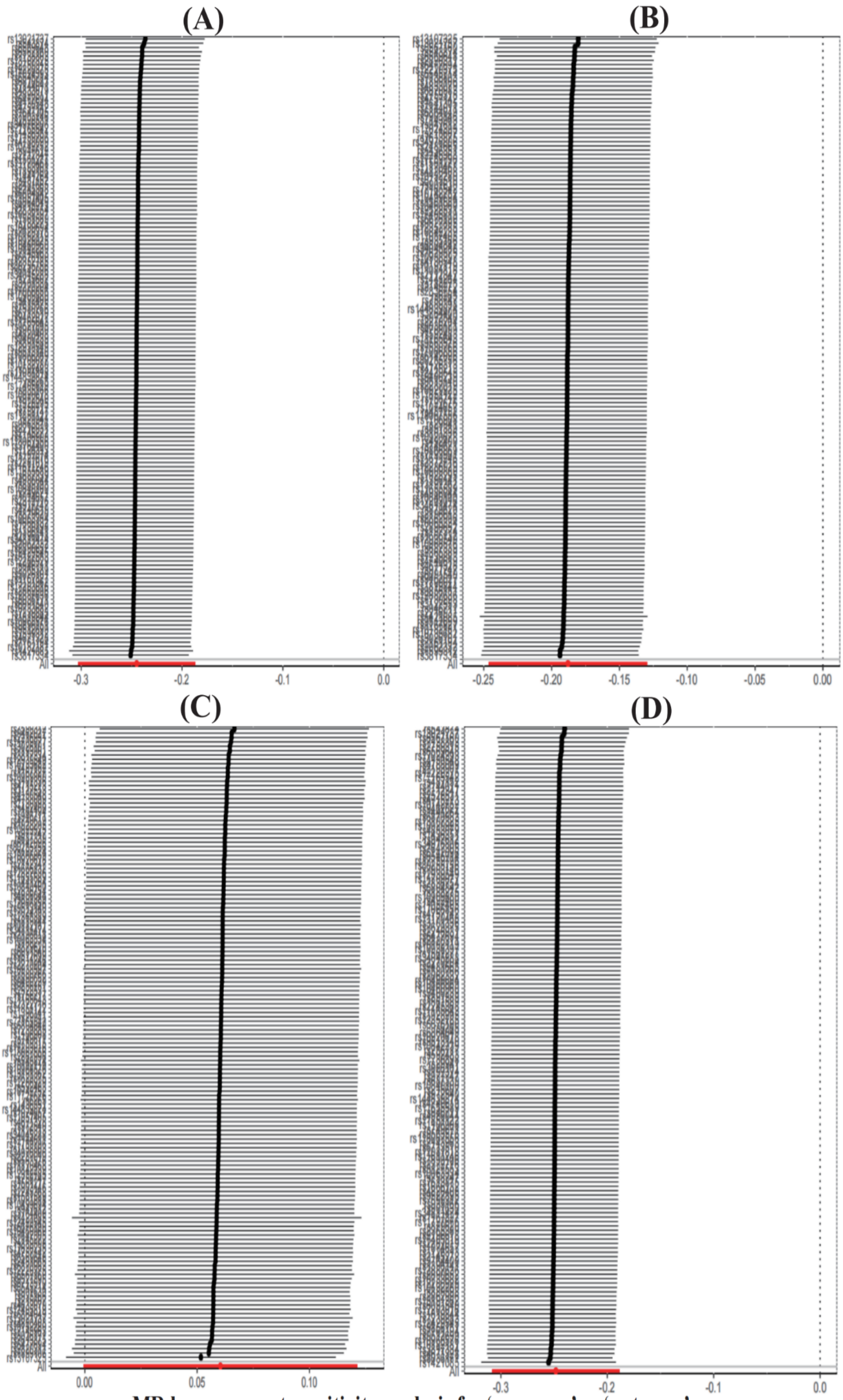

MR leave-one-out sensitivity analysis for 'exposure'on 'outcome'

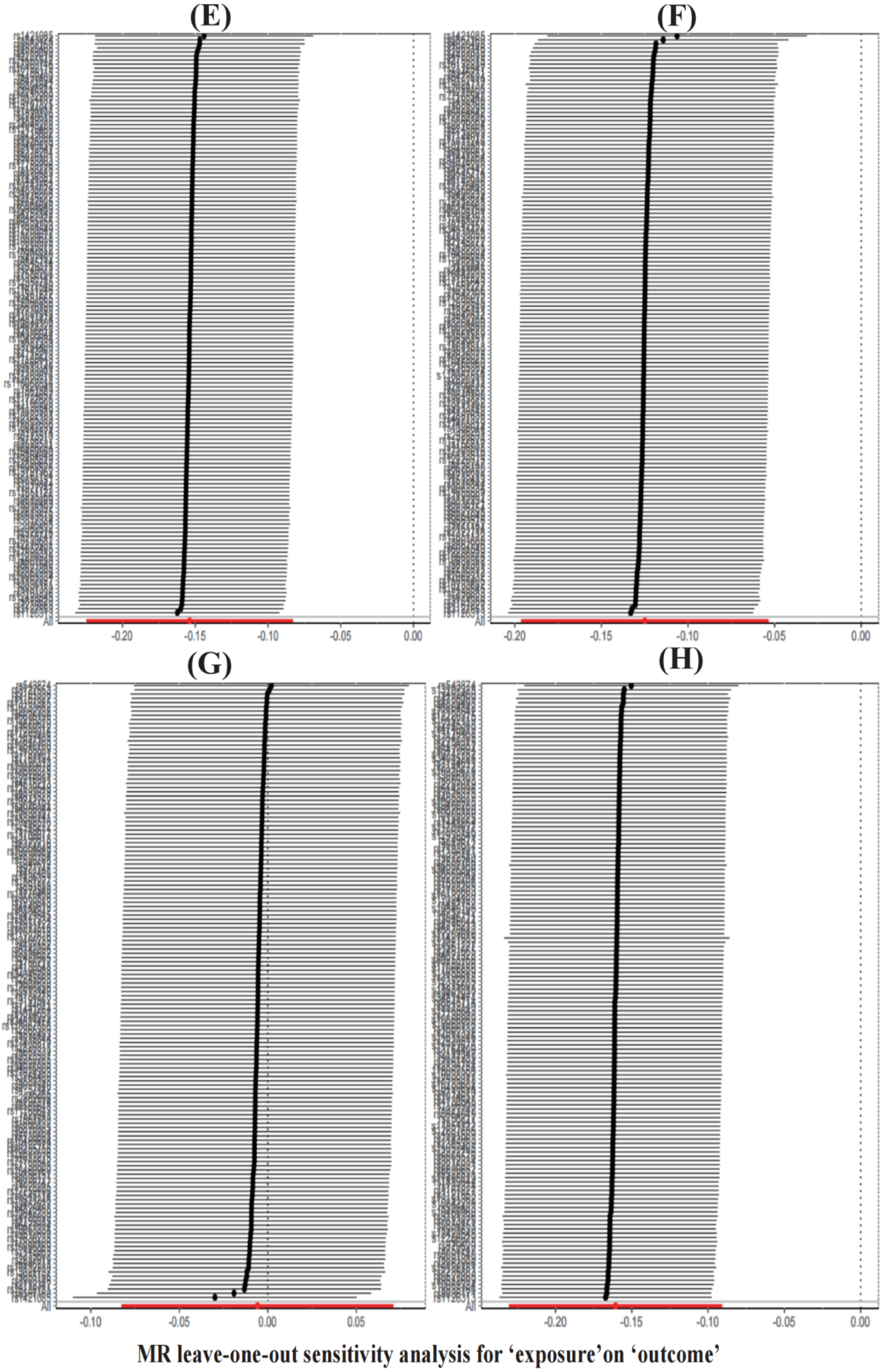

Supplementary Figure 19. Leave-one-out analyses of the association between BMI and LVEDV (A), LVESV (B), LVEF (C), LVSV (D), RVEDV (E), RVESV (F), RVEF (G), RVSV (H).

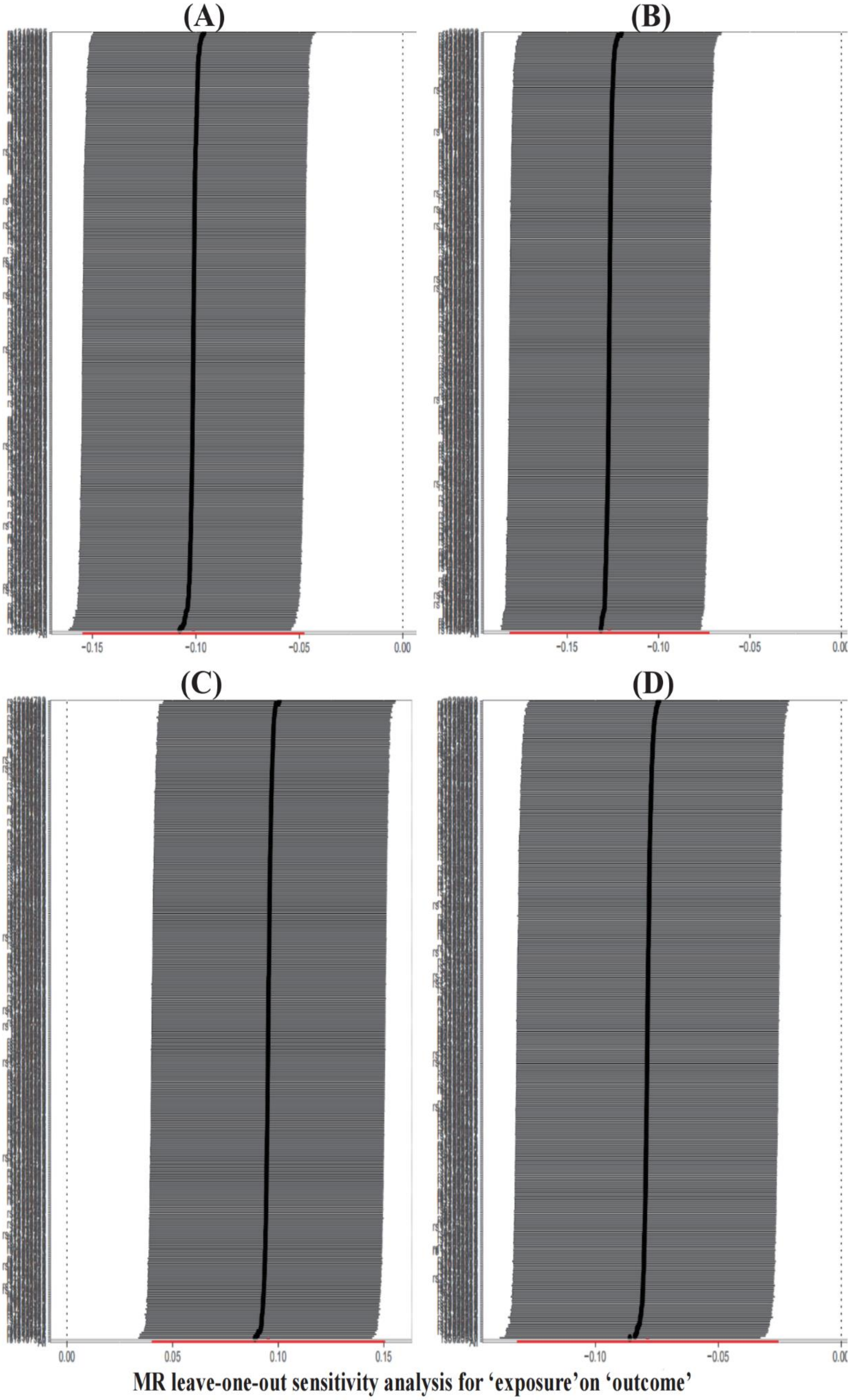

MR leave-one-out sensitivity analysis for 'exposure' on 'outcome'

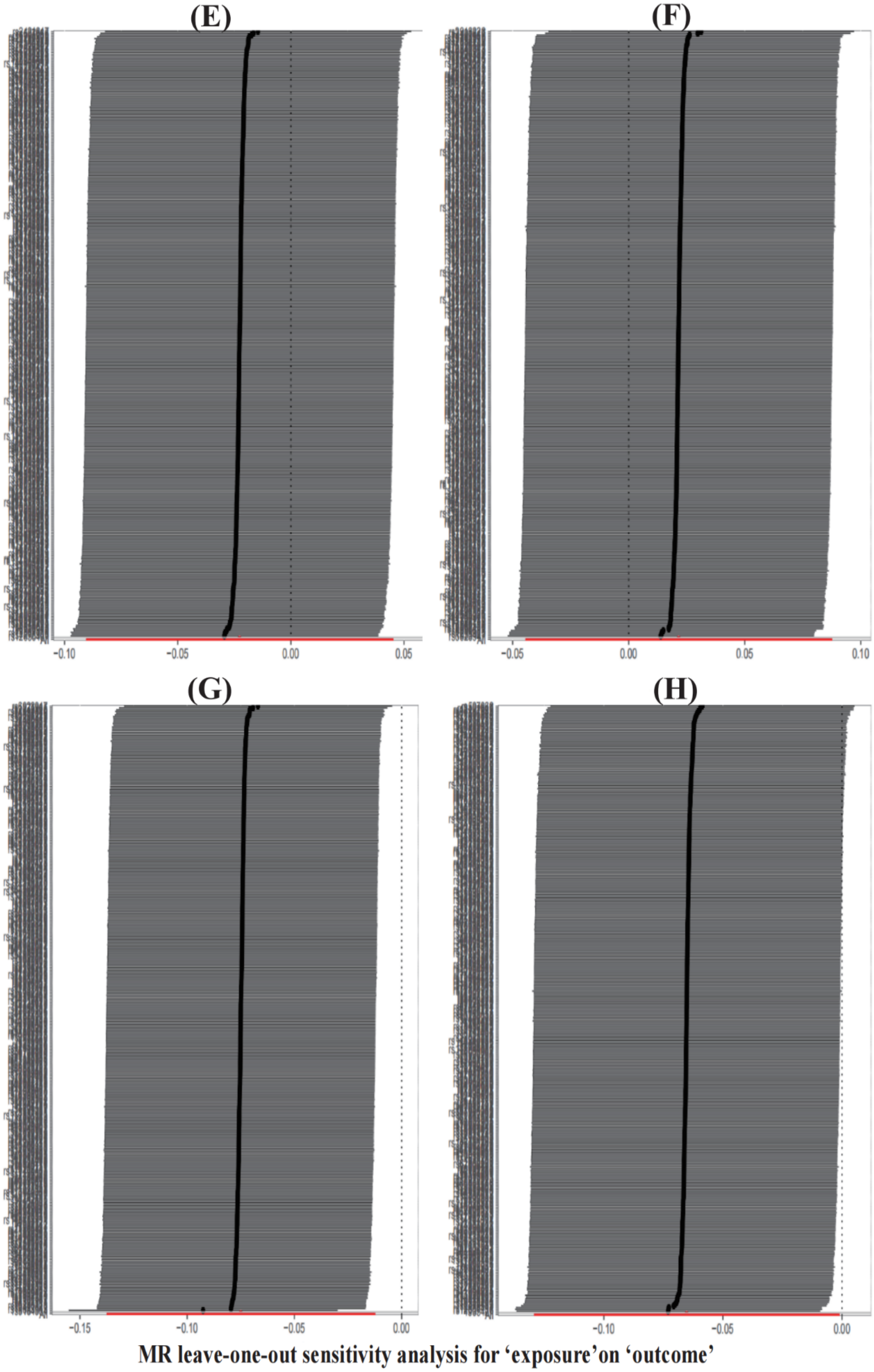

Supplementary Figure 20. Leave-one-out analyses of the association between DBP and LVEDV (A), LVESV (B), LVEF (C), LVSV (D), RVEDV (E), RVESV (F), RVEF (G), RVSV (H).

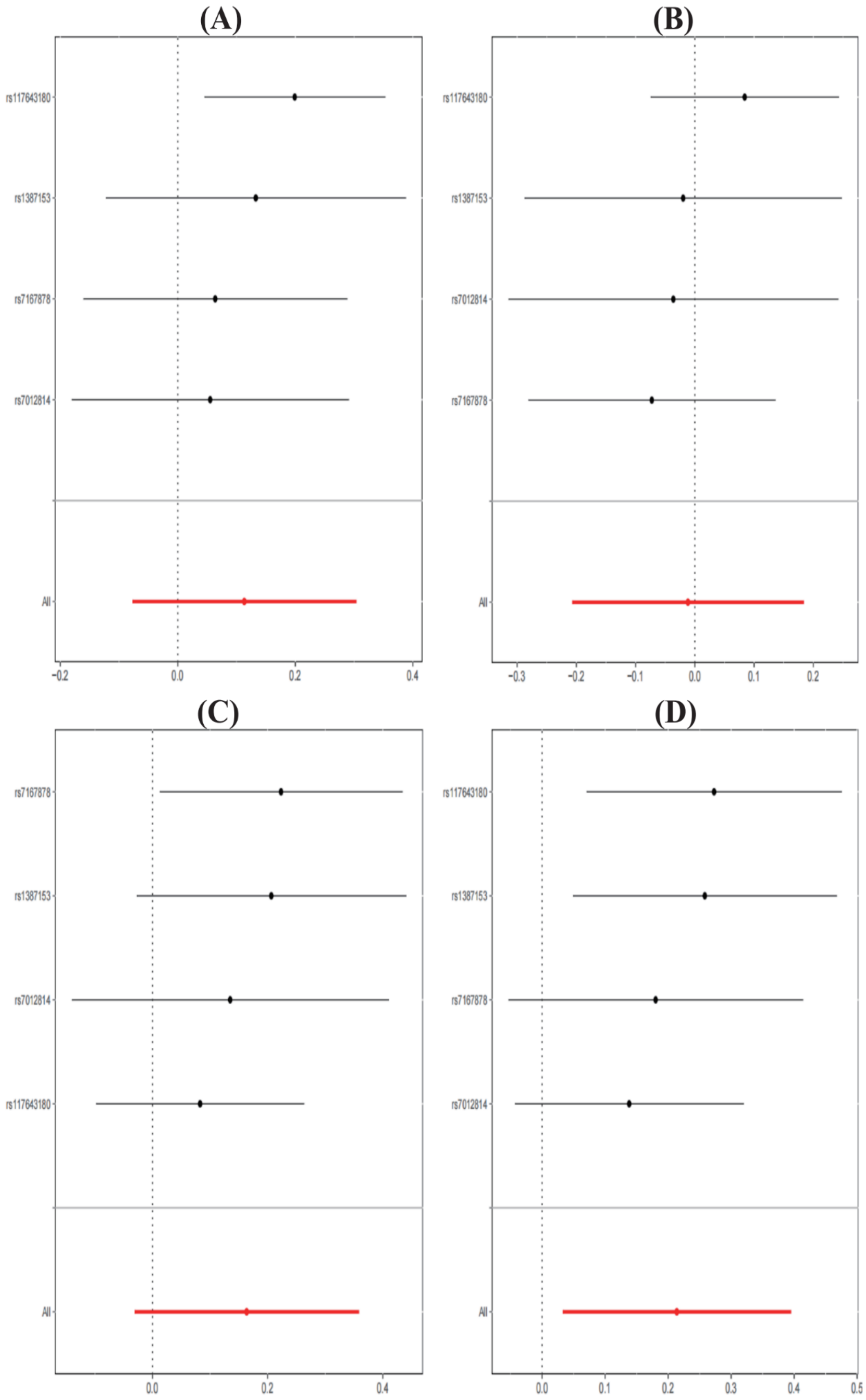

MR leave-one-out sensitivity analysis for 'exposure' on 'outcome'

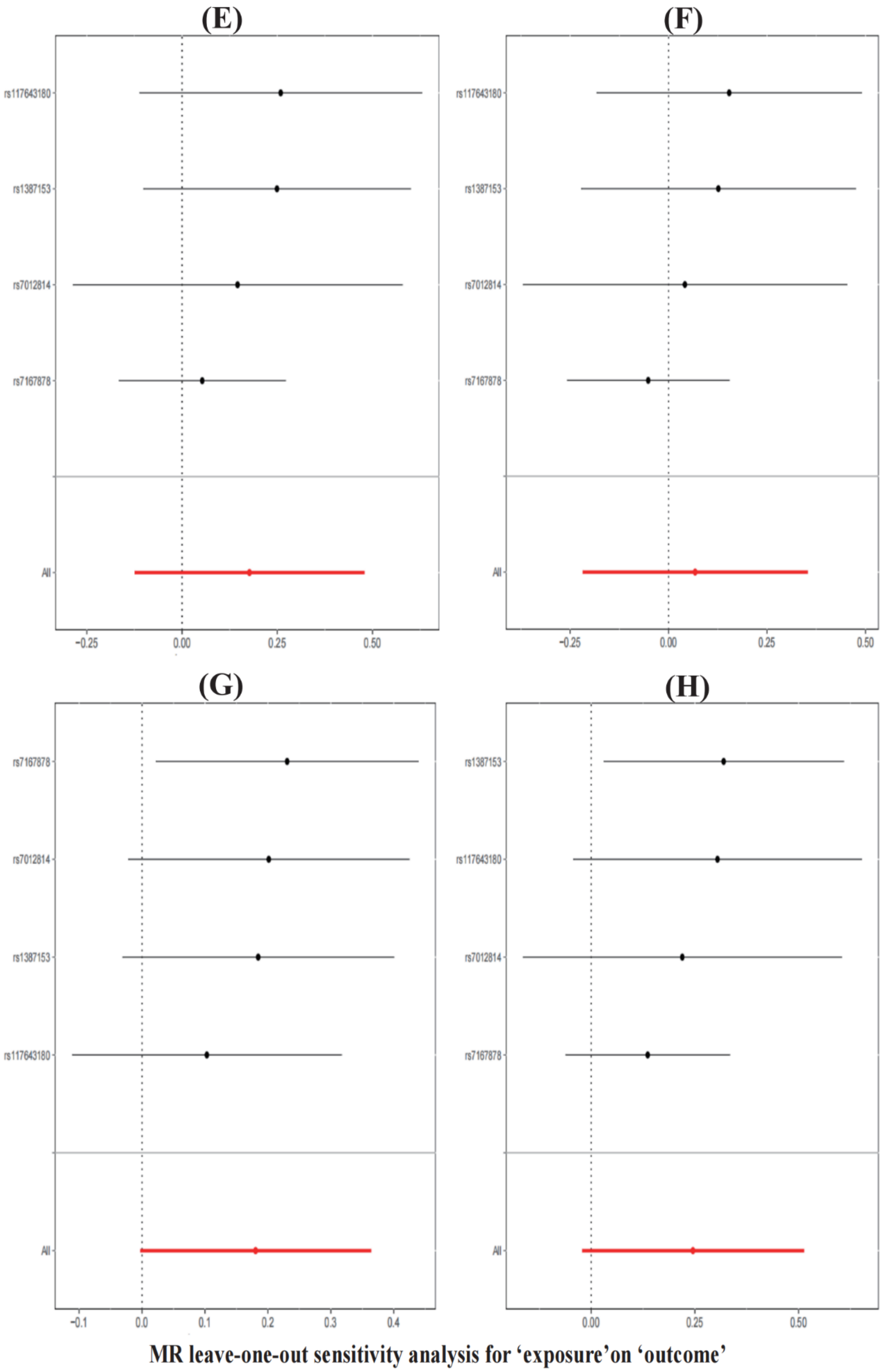

Supplementary Figure 21. Leave-one-out analyses of the association between IFCadjBMI and LVEDV (A), LVESV (B), LVEF (C), LVSV (D), RVEDV (E), RVESV (F), RVEF (G), RVSV (H).

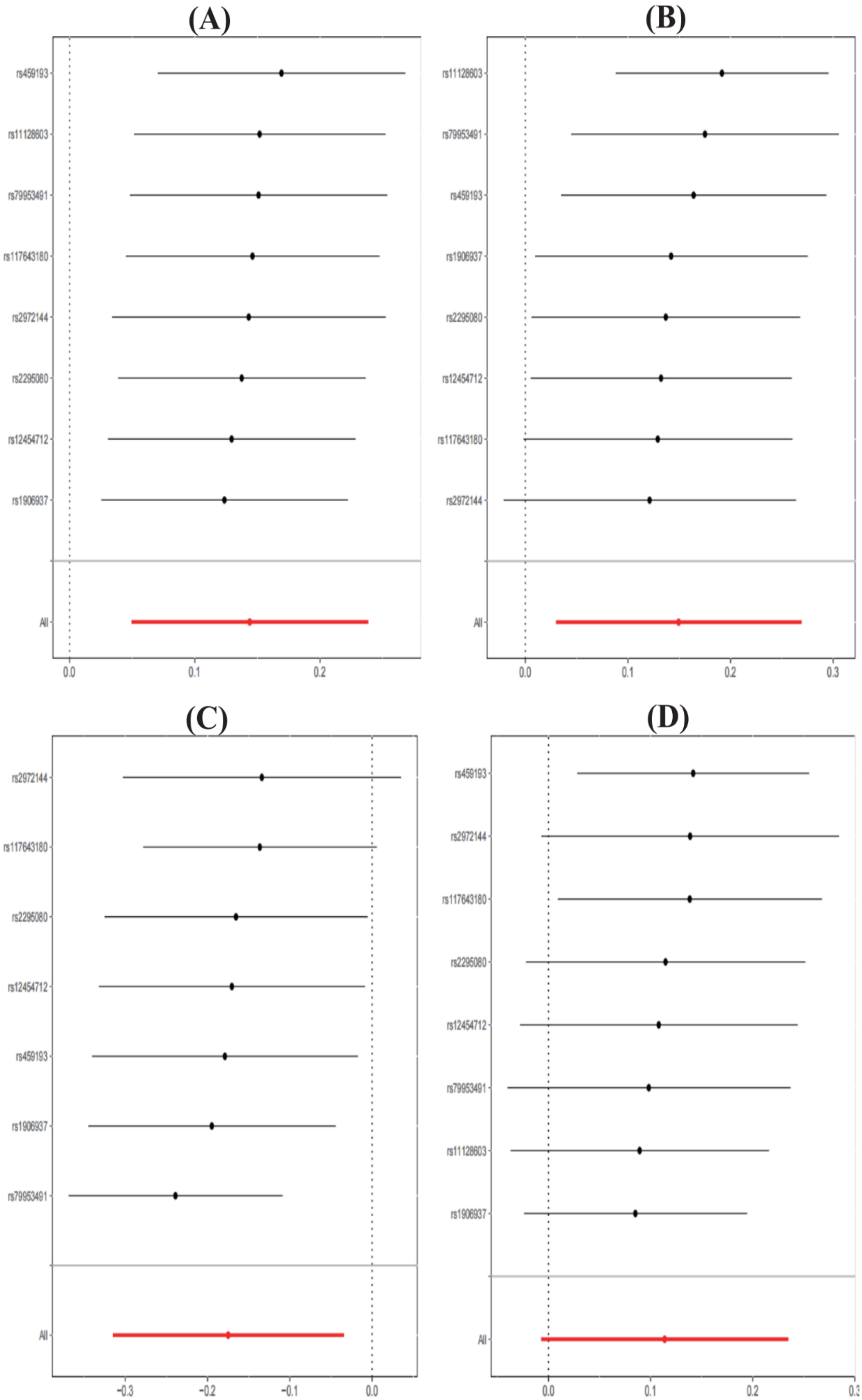

MR leave-one-out sensitivity analysis for 'exposure' on 'outcome'

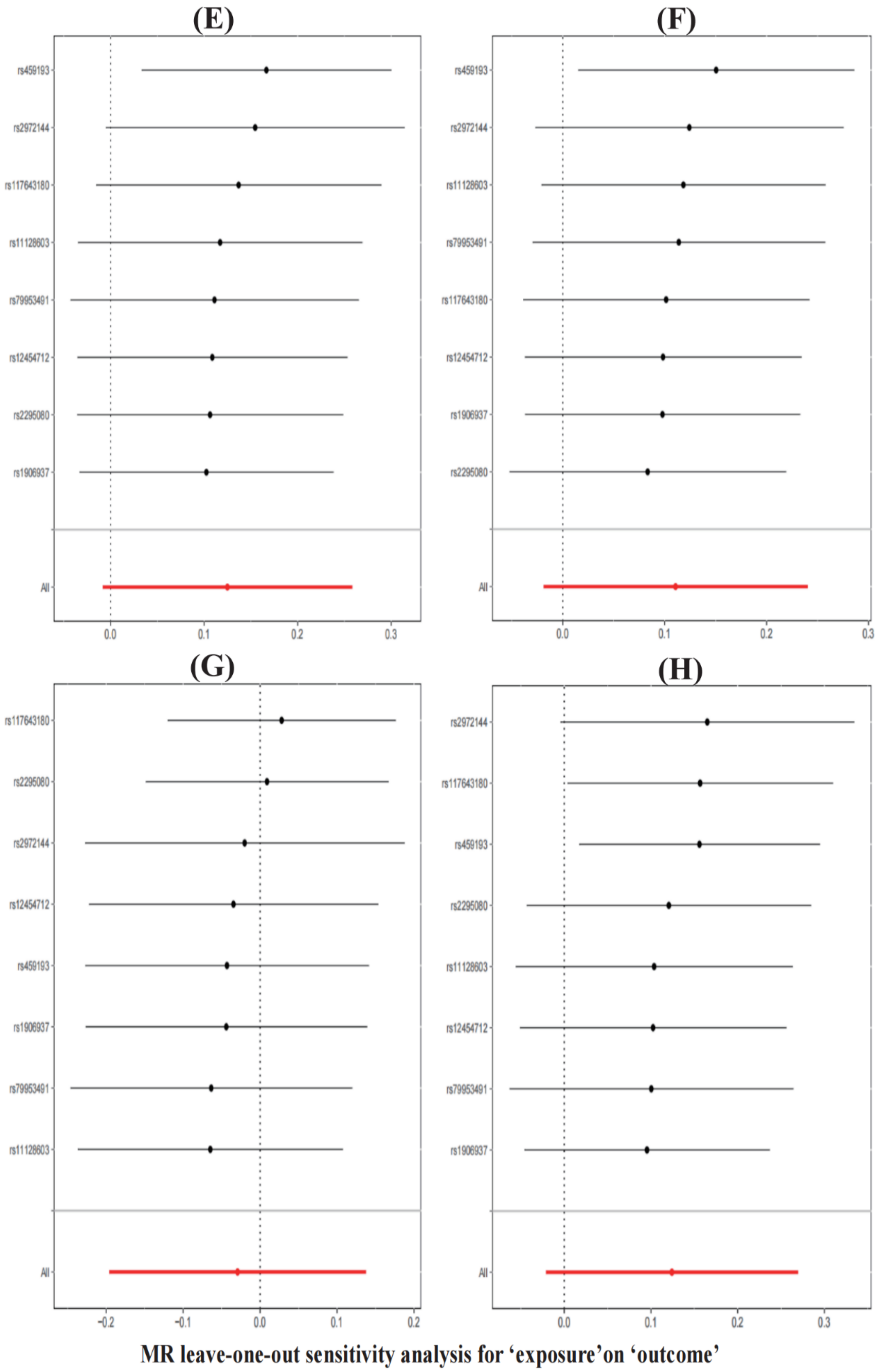

Supplementary Figure 22. Leave-one-out analyses of the association between ISIadjBMI and LVEDV (A), LVESV (B), LVEF (C), LVSV (D), RVEDV (E), RVESV (F),RVEF (G), RVSV (H).

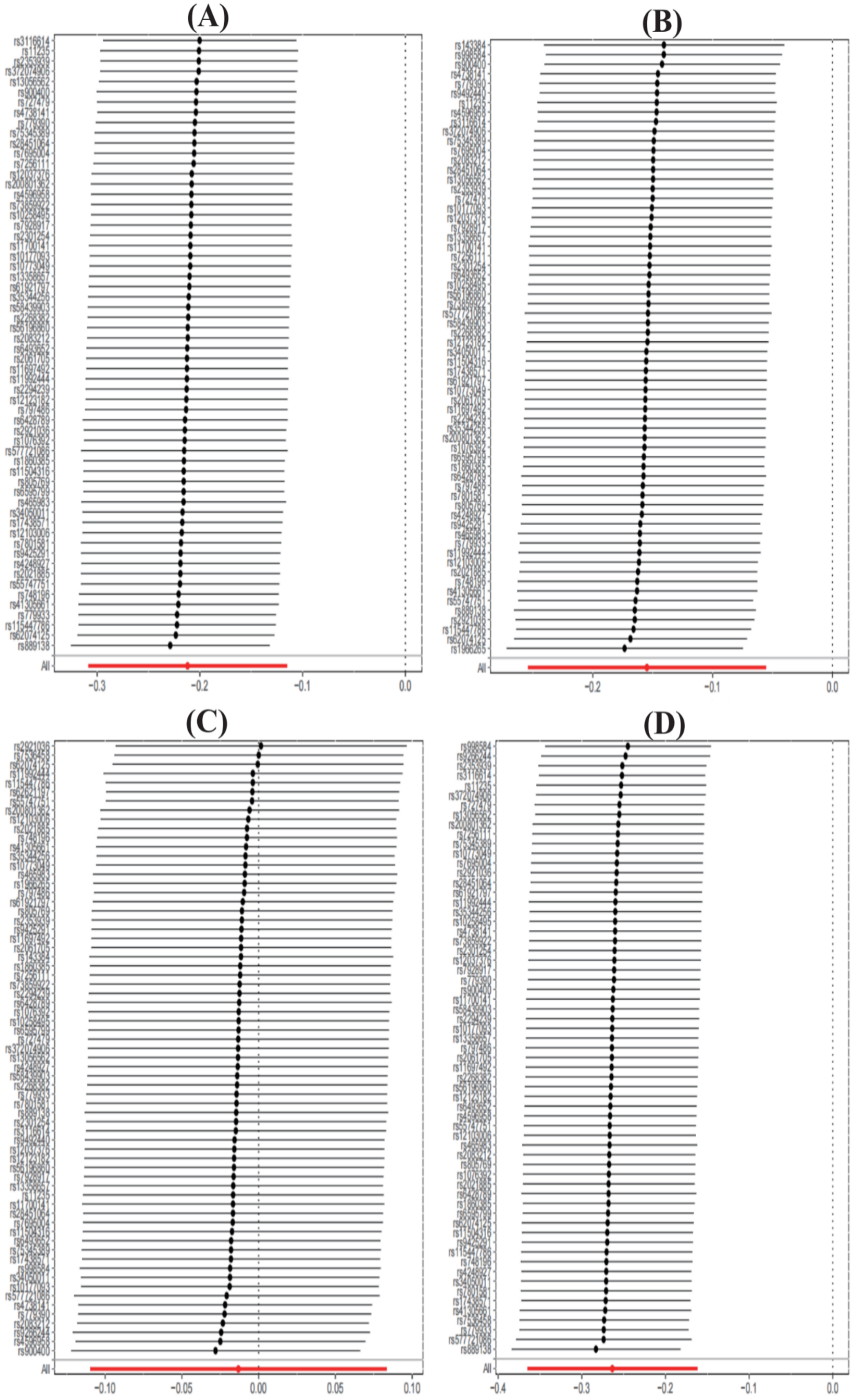

MR leave-one-out sensitivity analysis for 'exposure'on 'outcome'

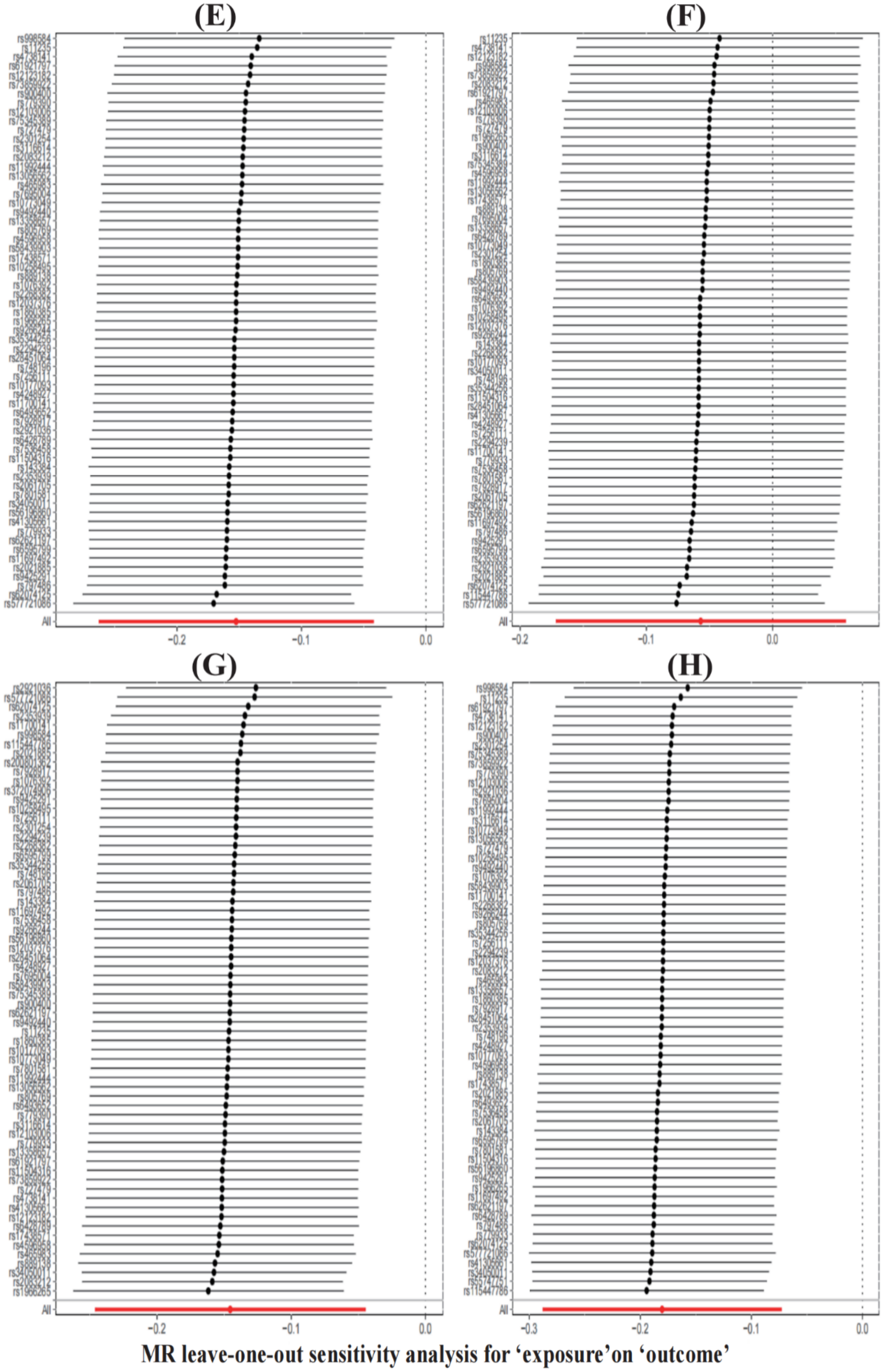

Supplementary Figure 23. Leave-one-out analyses of the association between WHRadjBMI(Man) and LVEDV (A), LVESV (B), LVEF (C), LVSV (D), RVEDV (E), RVESV (F), RVEF (G), RVSV(H).
